# NPM and NPM-MLF1 interact with chromatin remodeling complexes and influence their recruitment to specific genes

**DOI:** 10.1371/journal.pgen.1008463

**Published:** 2019-11-01

**Authors:** Anaïs Darracq, Helen Pak, Vincent Bourgoin, Farah Zmiri, Graham Dellaire, El Bachir Affar, Eric Milot

**Affiliations:** 1 Maisonneuve Rosemont Hospital Research Center, CIUSSS Est de l’Île de Montréal, boulevard l’Assomption, Montreal, Quebec, Canada; 2 Molecular Biology Program, University of Montreal, Montreal, Quebec, Canada; 3 Departments of Pathology and Biochemistry & Molecular Biology, Dalhousie University, Halifax, Nova Scotia, Canada; 4 Department of Medicine, University of Montreal, Boulevard Edouard-Montpetit, Montreal, Quebec, Canada; UNITED STATES

## Abstract

*Nucleophosmin (NPM1)* is frequently mutated or subjected to chromosomal translocation in acute myeloid leukemia (AML). NPM protein is primarily located in the nucleus, but the recurrent NPMc+ mutation, which creates a nuclear export signal, is characterized by cytoplasmic localization and leukemogenic properties. Similarly, the NPM-MLF1 translocation product favors the partial cytoplasmic retention of NPM. Regardless of their common cellular distribution, NPM-MLF1 malignancies engender different effects on hematopoiesis compared to NPMc+ counterparts, highlighting possible aberrant nuclear function(s) of NPM in NPMc+ and NPM-MLF1 AML. We performed a proteomic analysis and found that NPM and NPM-MLF1 interact with various nuclear proteins including subunits of the chromatin remodeling complexes ISWI, NuRD and P/BAF. Accordingly, NPM and NPM-MLF1 are recruited to transcriptionally active or repressed genes along with NuRD subunits. Although the overall gene expression program in NPM knockdown cells is similar to that resulting from NPMc+, NPM-MLF1 expression differentially altered gene transcription regulated by NPM. The abnormal gene regulation imposed by NPM-MLF1 can be characterized by the enhanced recruitment of NuRD to gene regulatory regions. Thus, different mechanisms would orchestrate the dysregulation of NPM function in NPMc+- *versus* NPM1-MLF1-associated leukemia.

## Author summary

NPMc+ mutation is the most common mutation in acute myeloid leukemia (AML) with prevalence in one third of all AML cases. NPM can also be involved in leukemogenic translocation including the t(3;5)(q25;q34) NPM-MLF1 translocation, which is associated to bad clinical course but remains poorly defined. We are reporting that NPM and the leukemogenic NPM-MLF1 play central role in chromatin organization and gene regulation in hematopoietic cells. A proteomic analysis provided the evidence that NPM and NPM-MLF1 are interacting with the chromatin remodeling complexes NuRD, P/BAF and ISWI in hematopoietic cells. The NPM nuclear depletion, such as imposed by the leukemogenic NPMc+ mutation, or the expression of NPM-MLF1 favors the uncontrolled recruitment of the CHD4/NuRD to chromatin and the abnormal regulation of NPM-target genes. Our results suggest that the abnormal gene regulation forced by NPM-MLF1 is different than the loss of nuclear function imposed by NPMc+, and it can be characterized by the enhanced recruitment of CHD4/NuRD to genes. Thus, NPM-MLF1 is likely to promote hematopoietic malignancies by disruption of gene regulation imposed by the NuRD activity.

## Introduction

Dysregulation of tumor suppressors and oncogenes can influence gene expression either directly or indirectly by altering the structure of chromatin and/or DNA methylation patterns, thereby reshaping the epigenome with consequences for a myriad of cellular functions. This phenomenon is particularly well described in acute myeloid leukemia (AML) [1, 2], where characteristic mutations or chromosomal translocations frequently affect the function of transcriptional regulators. For example, the frameshift mutation of *NPM1* exon 12 that promotes *de novo* creation of a nuclear export signal and promotes NPM cytoplasmic accumulation (NPMc+) [3], influences the transcriptome in hematopoietic cells [4, 5]. NPMc+ is detected in one third of AML cases and distinctively marks ~60% of cytogenetically normal AML (CN-AML; [3]). In addition, *NPM1* locus deletion or translocations (*e*.*g*., NPM-MLF1, NPM-ALK and NPM-RARα) are associated with many hematological malignancies including AML [6]. *NPM1* is one of the most commonly mutated/rearranged genes in hematological malignancies.

Determination of the mechanisms underlying the pathological effects of NPM mutation, rearrangement or deletion is complicated by the multiple cellular functions exerted by this factor [7–11]. Evidence for the participation of NPM in gene regulation is mainly supported by results indicating that it can interact with transcription factors [6, 12] and cofactors including the chromatin remodeler BRG1 [8] and histone modifiers such as HDAC1 and HDAC2 [10, 11]. However, it remains to be determined whether these interactions reflect the protein chaperone function of NPM [9, 13, 14] or the result of direct or indirect transcriptional regulation elicited by mutant NPM. Similarly, mechanistic details of how mutant NPM might alter transcription regulation remain to be fully elucidated.

The homodimerization capacity of NPM is maintained in NPMc+, thus favoring cytoplasmic localization of the native, non-mutated form of NPM [3]. Since NPM is predominantly located in the nucleus, the cytoplasmic relocalization imposed by NPMc+ creates a deficit in NPM nuclear function thereby promoting leukemia development [15]. This is supported by transcriptomic analyses of bone marrow cells from healthy donors *vs* AML patients, which demonstrate that multiple genes are deregulated in NPMc+ AML [4, 5]. However, the genetic and epigenetic heterogeneity that characterizes AML has hampered the investigation of precise mechanisms responsible for aberrant transcriptional regulation in NPMc+ leukemia cells.

The translocation product NPM-MLF1 retains the capacity to interact with NPM, and the resulting ‘heterodimer’ partially accumulates in the cytoplasm [16]. Thus, it was proposed that like NPMc+, NPM-MLF1 would promote leukemia through the loss of NPM nuclear function [3]. However, NPMc+ and NPM-MLF1 exert different effects on hematopoietic cells. Unlike NPMc+, which is associated with “frank leukemia” development and is a good prognostic marker, NPM-MLF1 is associated with pre-leukemic MDS (myelodysplastic syndrome) and poor prognosis AML [17]. Given the differential effects on hematopoietic development and the partial nuclear localization of the NPM-MLF1 fusion protein, it is possible that transcriptional programs are altered by a gain-of-function imposed by nuclear NPM-MLF1. Thus, defining the molecular mechanism(s) influenced by the oncoproteins NPMc+ and NPM-MLF1 would have important implications for targeted therapy, particularly for poor prognosis NPM-MLF1 AML.

We sought to determine how NPM and NPM-MLF1 influence the transcriptome of hematopoietic cells and assess whether the effect of NPM-MLF1 expression could reflect the effect of the absence of NPM and/or expression of NPMc+. NPM and NPM-MLF1 proteomic analysis performed in hematopoietic cells led to the identification of novel protein interactions involving NPM and/or NPM-MLF1. Prominent among these interactions were those involving several subunits of the chromatin remodeling complexes NuRD (nucleosome remodeling and deacetylase) [18], BAF, and/or PBAF (Polybromo-associated BAF) [19, 20], as well as ISWI (Imitation-SWItch) complexes [21, 22], suggesting the direct implication of NPM and NPM-MLF1 in shaping the epigenome of hematopoietic cells. Similar to previous studies of NPMc+ AML patient samples, our transcriptomic analysis indicates that NPM knockdown (NPM^KD^) elicits an aberrant gene expression signature. Mechanistically, we demonstrate that NPM promotes the targeted recruitment of NuRD complex to specific genes to regulate their expression, which is significantly impacted by NPM^KD^. We demonstrate that residual nuclear NPM-MLF1 directly binds chromatin and can enhance recruitment of NuRD to gene regulatory regions. Based on our results, we propose that the NPM-MLF1 translocation contributes to leukemogenesis by the modification of gene expression, which can be associated to the altered recruitment of NuRD.

## Results

### Characterization of the NPM and the NPM-MLF1 protein interactomes in hematopoietic cells

Western blot assays of Flag-HA-NPM (FH-NPM), FH-NPMc+ and FH-NPM-MLF1 cell lysates indicated that expression of the transgenic proteins was less than 50% of the endogenous NPM protein in all K562 clones (respectively, K562/NPM, K562/NPMc+ and K562/NPM-MLF1; S1A and S1B Fig), hence precluding artefactual protein-protein interactions due to overexpression. The molecular weight of the FH-NPM and FH-NPMc+ is ~40 kDa and that of FH-NPM-MLF1 is ~70 kDa. Low transgene expression was also indicated by RT-qPCR (S1C Fig). Since the levels of mRNA are not strictly correlated with transgene protein levels, post-transcriptional mechanisms are likely to be important to control the accumulation of these proteins. The characterization of these models indicated that expression of the transgenes had no significant effect on cell cycle, apoptosis (S1D Fig) or cell proliferation in exponentially growing cultures (S1E Fig), even though the NPMc+ expressing cells escaped growth inhibition when approaching confluence. In any case, during log phase growth, the low-level expression of transgenes does not substantially influence proliferation of the different clones, thus allowing comparative analyses.

Immunostaining detection of FH-NPM, FH-NPMc+ and FH-NPM-MLF1 indicated that, as expected [15, 16], FH-NPM was mostly located in the nucleus with strong accumulation in nucleoli; whereas FH-NPM-MLF1 rather than being located to nucleoli, was found in nuclear puncta and exhibited an increased localization in the cytoplasm (S1F Fig). Similarly, a portion of FH-NPMc+ also relocated to the cytoplasm (S1F Fig). This is consistent with the previously described localization of NPMc+ in the patient-derived leukemic cells OCI-AML3 (S1G Fig), which express NPMc+ as a result of oncogenic mutation [23]. Finally, in both NPMc+ and NPM-MLF1 expressing cells, the endogenous NPM was detected in cytoplasmic and nuclear fractions (S1H and S1I Fig).

**Fig 1:**
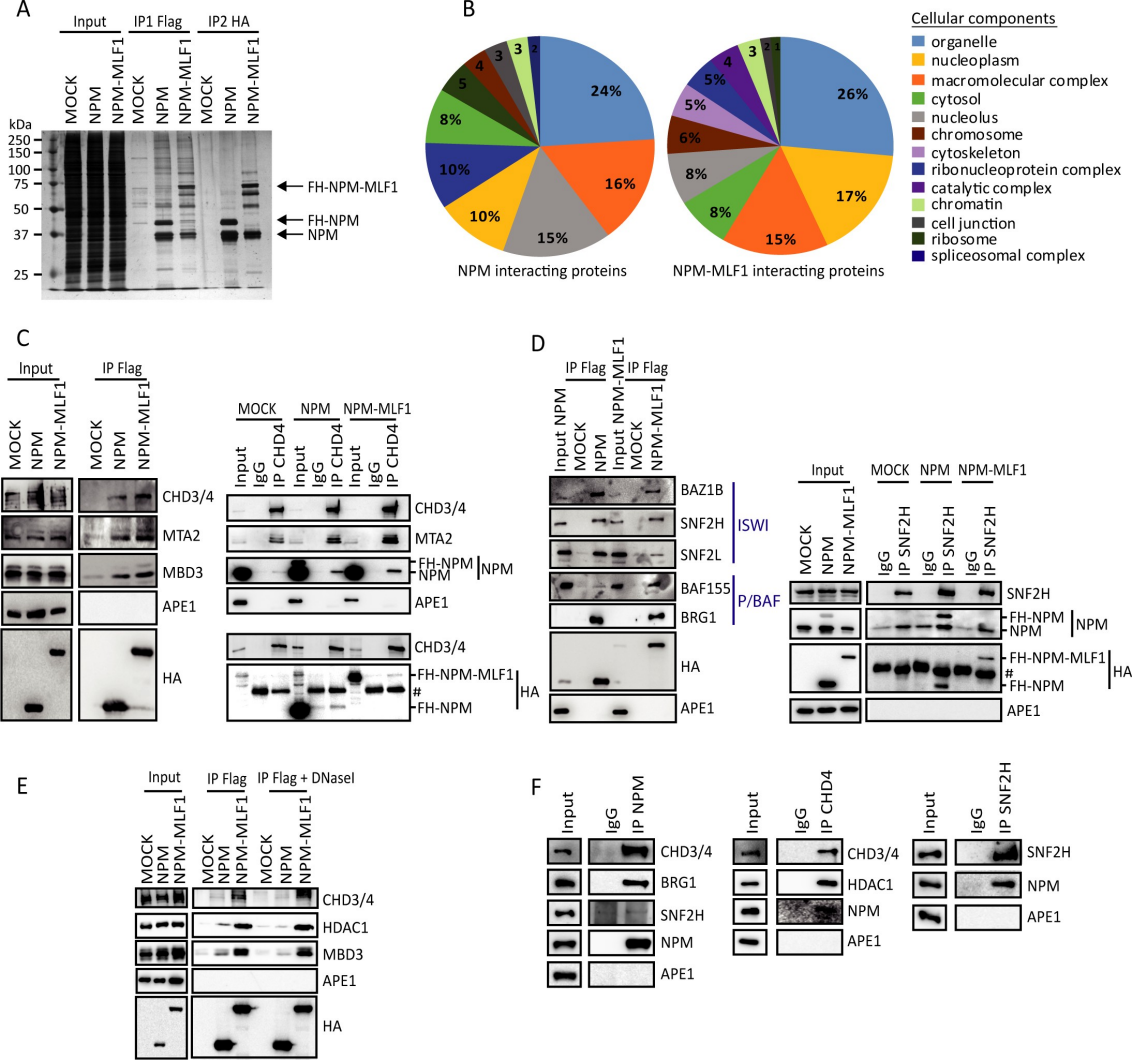
NPM and NPM-MLF1 interact with chromatin remodeling complexes. **A**) Flag-HA tandem immunoaffinity purification; nuclear extract from MOCK, NPM or NPM-MLF1 expressing cells were used for sequential immunoaffinity purification and a fraction of the different purification steps was loaded and detected by silver staining; Input: nuclear extracts; IP1 Flag: Flag purification; IP2 HA: HA purification; arrows indicate the bands corresponding to FH-NPM-MLF1, FH-NPM and the endogenous NPM; protein molecular weights are indicated on the left side of the panel; **B**) LC-MS/MS data analysis made with STRING database; NPM and NPM-MLF1 interacting partners are classified according to cellular component organization; **C**, **D**) Co-immunoprecipitation assays; K562 nuclear extracts of MOCK, NPM or NPM-MLF1 expressing cells were immunoprecipitated (IP) with Flag, CHD4, SNF2H antibodies or isotype-matched immunoglobulins (IgG); the antibodies used for Western blots are indicated on the right side of the panels; antibodies were specific for NuRD (panel **C**) or ISWI and P/BAF (panel **D**) complex; APE1 was used as negative control; **E**) Immunoprecipitation assay with Flag antibodies; K562 nuclear extracts of MOCK, NPM or NPM-MLF1 were treated with 10 μg/mL of DNaseI; **F**) Co-immunoprecipitation experiments were performed with NPM, BRG1, SNF2H antibodies or isotype-matched immunoglobulin (IgG) in HEL WT cell nuclear extracts. The proteins detected by Western blot are indicated on the right side of the panels.

The prevalent model to explain how NPMc+ and NPM-MLF1 can promote leukemia is related to their capacity to relocate NPM to the cytoplasm and hence, favor nuclear depletion of NPM [3, 15, 16]. Although the cytoplasmic accumulation of NPMc+ was demonstrated to be required for its leukemogenic capacity [24], NPMc+ and NPM-MLF1 might impose a nuclear gain-of-function due to the displacement of NPM-interacting proteins within the nucleus and/or by modifying cofactor recruitment to specific genes. The latter is particularly conceivable for NPM-MLF1, since MLF1 overexpression and nuclear localization were reported to affect regulation of specific genes [25, 26]. To test this hypothesis, we defined the NPM and NPM-MLF1 interactomes in hematopoietic cells. The identification of nuclear proteins interacting with NPM and/or NPM-MLF1 was performed by tandem immunoaffinity purification (TAP-Tag) followed by LC-MS/MS analysis [27, 28]. TAP-tag purification was monitored by silver staining (Fig 1A), and the lists of potential interacting proteins are presented in S1 File. NPM and NPM-MLF1 interactomes were analyzed using STRING database (https://www.ncbi.nlm.nih.gov/pubmed/27924014; accessed on 20/08/2018) and the proteins were regrouped according to cellular components and biological processes (Table 1; Fig 1B and S2 Fig). In agreement with the IF analysis (S1F and S1I Fig), NPM interacts with many nucleolar proteins whereas NPM-MLF1 interacts mostly with nucleoplasmic factors (Fig 1B). Proteins associated with organelles, macromolecular complexes, cytosol, chromatin, and cell junctions are represented in similar proportions in the NPM and NPM-MLF1 interactomes. However, the percentage of proteins in catalytic complexes and cytoskeleton is enhanced in the NPM-MLF1 interactome, and the percentage of proteins involved in RNA splicing is larger in the NPM interactome (Fig 1B). Furthermore, biological process analysis suggests that NPM is primarily implicated in ribosomal function, consistent with its localization in nucleoli (S1F Fig). On the other hand, the NPM-MLF1 interactome was enriched in nucleoplasmic proteins including those involved in gene regulation. Thus, the comparative analysis of the interactomes of NPM and NPM-MLF1 indicates differences in proteins with nuclear functions and suggests that the oncogenic NPM-MLF1 fusion protein may affect distinct cellular mechanisms due to alteration in its affinity (vs NPM) for specific nuclear complexes. Interestingly, our interactome analysis revealed that both NPM and NPM-MLF1 are associated with chromatin organization factors since many peptides corresponding to subunits of the remodeling complexes NuRD, P/BAF and the ISWI family [18, 20, 29, 30], were identified by LC-MS/MS (Table 1 and S2 Fig). According to the number of peptides and subunits detected for NuRD or P/BAF complex, the NPM interactions with these remodeling complexes are likely to be less frequent or less stable than the corresponding interactions with NPM-MLF1. Indeed, the number of NPM peptides detected by the LC-MS/MS analysis was greater for the NPM TAP-Tag than for the NPM-MLF1 TAP-Tag analysis (Table 1), and no apparent difference was noted for NPM *vs* NPM-MLF1 interactions with the ATPases SNF2H (SMARCA5) and SNF2L (SMARCA1), which are alternatively included in the ISWI-type complexes [22]. The complete lists of factors involved in transcriptional regulation identified by the LC-MS/MS analysis are presented in S2 File. Many of these potential direct or indirect interactions (hereafter referred to as interactions) were subsequently confirmed by co-immunoprecipitation (co-IP) with Flag, CHD4 (NuRD ATPase) and SNF2H (ISWI ATPase) antibodies, hence further supporting the interactions between NPM-MLF1 and subunits of the NuRD, P/BAF and ISWI-type complexes (Fig 1C and 1D). The co-IP were performed on nuclear extracts that contain deniable traces of DNA (S3 Fig) and DNaseI treatment did not affect NPM and NPM-MLF1 protein interactions with CHD3/CHD4, MTA2, MBD3 (Fig 1E). Finally, since we also observed these interactions in the erythroleukemia cell line HEL (Fig 1F), we conclude that (i) interactions between NPM and CHD4, BRG1 or SNF2H are not cell line-dependent; and (ii) the endogenous NPM can interact with these remodeling complexes in the absence of the transgenic NPM or NPM-MLF1 protein.

**Table 1:** Immunoaffinity purification and identification of FH-NPM and FH-NPM-MLF1 interacting proteins from K562 cells, by LC-MS/MS analysis.

**NPM associated factors**					
**Gene symbol**	**Nb unique peptides**	**Nb peptides total**	**AVG**	**Coverage**	**Complex**
NPM	49	704	3.4781	72.4%	-
HDAC1	1	2	2.7785	2.5%	NuRD
MTA1	1	1	1.5869	1.4%	NuRD
GATAD2A	2	2	2.7539	3.6%	NuRD
SMARCC1 (BAF155)	13	13	2.4901	12.4%	P/BAF
SMARCC2 (BAF170)	5	5	2.7647	5.4%	P/BAF
ACTL6A	3	3	3.2014	10.7%	P/BAF
SMARCB1 (SNF5)	2	2	2.8289	7.8%	P/BAF
SMARCD2 (BAF60B)	2	2	3.3591	5.3%	P/BAF
SMARCD1 (BAF60A)	1	1	3.407	2.5%	P/BAF
SMARCA4 (BRG1)	1	1	2.6197	0.7%	P/BAF
BAF180 (PBRM1)	1	1	3.6717	1.1%	P/BAF
DDX21	27	34	3.0167	35%	ISWI
SMARCA5 (SNF2H)	23	26	2.7362	22.2%	ISWI
SMARCA1 (SNF2L)	9	9	2.6112	5.8%	ISWI
MYBBP1A	7	7	2.6662	5.3%	ISWI
WSTF (BAZ1B)	5	5	2.4308	4.4%	ISWI
DEK	2	2	2.7281	8.3%	ISWI
**NPM-MLF1 associated factors**					
**Gene symbol**	**Nb unique peptides**	**Nb peptides total**	**AVG**	**Coverage**	**Complex**
NPM	30	202	3.3753	68%	-
CHD4	19	24	2.7103	9.7%	NuRD
HDAC1	12	13	2.8632	23.7%	NuRD
HDAC2	8	9	3.2925	14.5%	NuRD
RBBP4	8	9	2.8428	26.1%	NuRD
CHD3	7	8	2.8151	4.5%	NuRD
GATAD2A	5	5	2.7687	10.4%	NuRD
MTA2	4	5	3.166	8.8%	NuRD
MTA1	3	3	2.8883	4.9%	NuRD
MBD3	1	1	3.1199	4.8%	NuRD
GATAD2B	1	1	3.4446	4.2%	NuRD
BAF180 (PBRM1)	27	29	2.771	18.2%	P/BAF
SMARCC1 (BAF155)	13	14	2.8801	16.8%	P/BAF
SMARCA2 (BRM)	8	9	2.6807	6%	P/BAF
SMARCA4 (BRG1)	8	8	3.0084	6.4%	P/BAF
ARID2	7	7	2.7209	6.9%	P/BAF
SMARCC2 (BAF170)	6	6	3.615	7.4%	P/BAF
ACTL6A	3	3	3.1549	8.6%	P/BAF
BRD7	3	3	3.5831	9.8%	P/BAF
SMARCB1 (SNF5)	1	1	3.3006	3.6%	P/BAF
SMARCD1 (BAF60A)	1	1	2.9127	2.5%	P/BAF
SMARCE1 (BAF57)	1	1	3.303	2.9%	P/BAF
SMARCD2 (BAF60B)	1	1	3.2464	2.8%	P/BAF
SMARCE1	1	1	3.303	2.9%	P/BAF
BAF45A (PHF10)	1	1	2.4624	3.6%	P/BAF
BCL7C	1	1	2.2226	5.5%	P/BAF
BCL7A	1	1	1.8993	4.8%	P/BAF
SMARCA5 (SNF2H)	19	20	3.0813	18.5%	ISWI
BPTF	12	12	3.0648	5.6%	ISWI
SMARCA1 (SNF2L)	10	11	2.2798	7.5%	ISWI
DDX21	9	10	2.8947	15.1%	ISWI
WSTF (BAZ1B)	7	8	3.0102	7.1%	ISWI
MYBBP1A	2	2	2.7507	1.9%	ISWI

Gene symbols, number of unique and total peptides, corresponding average mass (AVG), percentage of coverage and known protein complexes are indicated. P/BAF: BAF and PBAF complexes.

**Fig 2:**
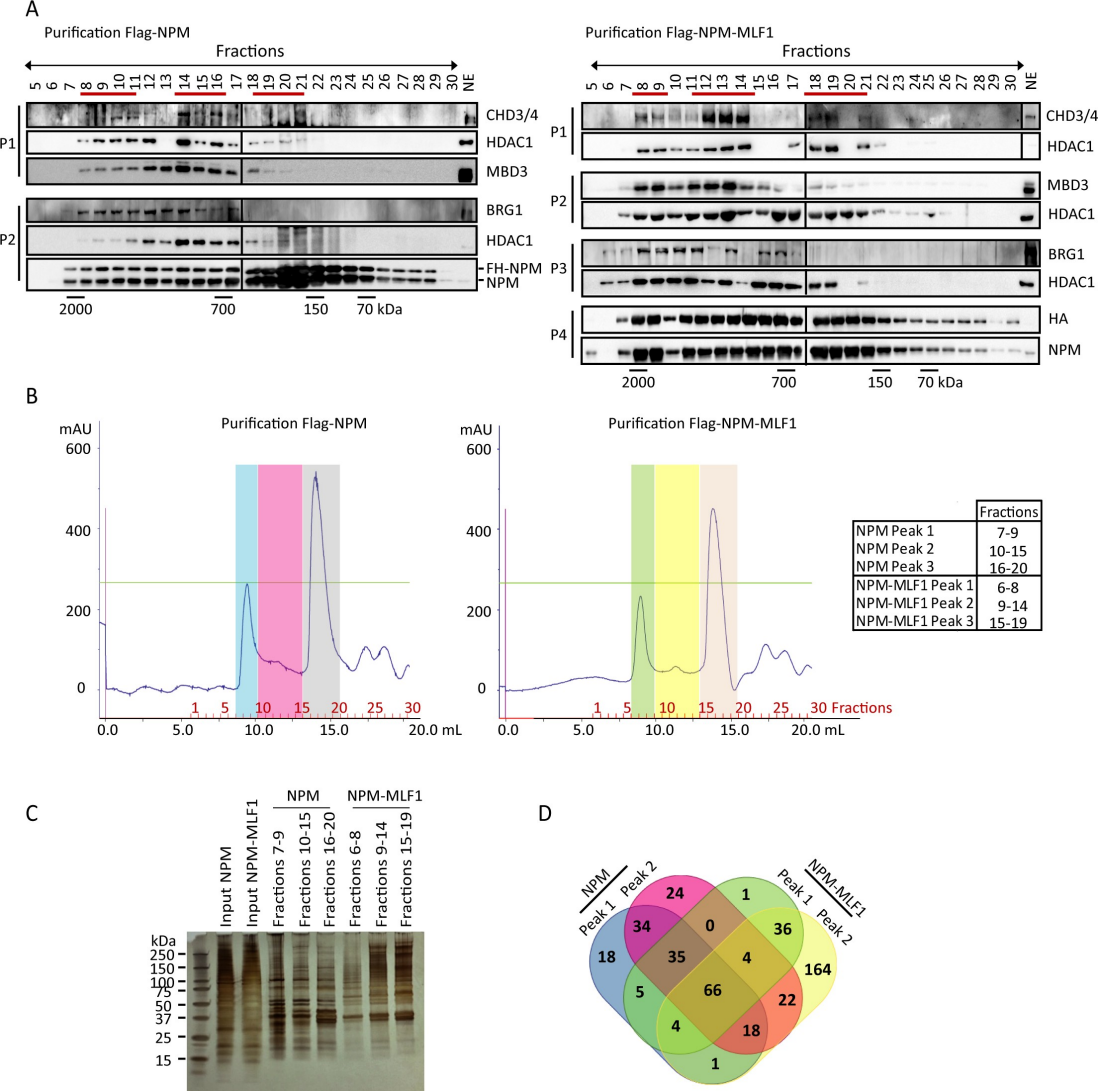
Characterization of NPM and NPM-MLF1 interacting complexes. **A**) Size-exclusion chromatography (FPLC); Flag-NPM and Flag-NPM-MLF1 affinity purified complexes were fractionated on Superose 6 10/300GL column; the antibodies used for Western blot are indicated on the right side of the panels; average fraction size in kDa is indicated under the panels; red lines above panels indicate NuRD subunits elution peaks; NE: nuclear extract; P1, P2, P3 and P4 refer to different purification experiments. **B)** Chromatograms of the Flag-NPM and Flag-NPM-MLF1 purifications after size-exclusion by FPLC. Fraction numbers are indicated at the bottom of the chromatograms. Fractions were regrouped according to the peaks (each with a distinct color) and were used for MS/MS analyses (NPM Peak 1: fractions 7–9; NPM Peak 2: fractions 10–15; NPM Peak 3: fractions 16–20; NPM-MLF1 Peak 1: fractions 6–8; NPM-MLF1 Peak 2: fractions 9–14; NPM-MLF1 Peak 3: fractions 15–19). **C)** Silver staining of the Inputs and regrouped fractions corresponding to each; Input: nuclear extracts. **D)** Venn diagram indicating the distribution of proteins detected in NPM peaks 1 and 2, NPM-MLF1 peaks 1 and 2. The number of proteins in each sections of the diagram is indicated.

We focused on the interactions of NPM and NPM-MLF1 with NuRD and P/BAF since the TAP-Tag results suggest that the subunits of these two complexes preferentially interact with NPM-MLF1 (*vs* NPM; Table 1), and both are critical regulators of transcription in hematopoietic cells. To determine the size and composition of the NuRD and P/BAF complex(es) associated with NPM and NPM-MLF1, we performed size exclusion FPLC on nuclear extracts from NPM- and NPM-MLF1-expressing cells. NPM protein eluted in multiple fractions, suggesting that it is included in different nuclear complexes (S4 Fig). Since the fractions whereby NPM was detected were the same in NPM- and NPM-MLF1-expressing cells (S4 Fig), we conclude that NPM-MLF1 expression does not substantially modify the endogenous NPM interactome. As predicted, NPM and NPM-MLF1 co-fractionated with the NuRD subunits CHD3/CHD4, HDAC1 and MBD3 as well as the P/BAF nucleosome remodeling ATPase BRG1 (S4 Fig). To further demonstrate complex formation, FPLC was also performed after Flag immunoaffinity purification of FH-NPM and FH-NPM-MLF1 nuclear extracts (Fig 2A). Size exclusion chromatography of Flag immunoaffinity purified proteins indicated that NPM and NPM-MLF1 co-elute with CHD3/CHD4, HDAC1, and MBD3 in different fractions regrouped in three distinct peaks. The NPM-NuRD and NPM-MLF1-NuRD peaks/complexes were regrouped in fractions around 2000 kDa, 750 kDa and 600 kDa (Fig 2A; S5A Fig). Nonetheless, the fractionation pattern of NPM-MLF1 was different from that of NPM since a large portion of the former was included in fractions ~2000 kDa to 750 kDa and had a distinctive increase of NuRD subunits and BRG1 in fractions near 1100 kDa (right panels; Fig 2A). Thus, the fusion protein NPM-MLF1 is likely to impose variations in composition of the remodeling complexes NuRD and P/BAF. To gain information regarding the effect of NPM-MLF1 on complex formation, we repeated the immunoaffinity purifications of FH-NPM and FH-NPM-MLF1 (Fig 2B and 2C) followed by size exclusion chromatography. The collected fractions were used for LC-MS/MS analysis in order to identify proteins co-eluting in distinct complexes. The FPLC fractions from 2000 kDa to 600 kDa were collected and divided in 3 groups according to peaks that were defined by the abundance of remodeling complex factors (see Fig 2B and 2C): (peak 1) fractions around 2000 kDa; (peak 2) fractions around 1100 kDa; and (peak 3) fractions around 750 kDa. The MS results are presented in S3 File and are summarized in Table 2 and Fig 2D. The comparative analysis of the MS results indicates that NuRD and ISWI subunits eluted primarily in ‘peak 2’ fractions of FH-NPM and FH-NPM-MLF1 samples. The subunits of the P/PAF are primarily in ‘peak 1’ of the FH-NPM sample but importantly, half of P/BAF subunits detected in the HF-NPM-MLF1 sample are primarily eluting in ‘peak 2’ instead of peak 1, thus suggesting the reorganization of P/BAF in NPM-MLF1 expressing cells. Based on these results, we next assessed the existence of a complex containing CHD4 and BRG1. Using co-IP procedure, we could not detect interaction between these nucleosome remodeling ATPases in the NPM or NPM-MLF1 cells (S5B Fig), thus suggesting the absence or low occurrence of complex(es) including both remodeling activities.

**Table 2:** LC-MS/MS analysis of size exclusion chromatography after immunoaffinity purification of FH-NPM or FH-NPM-MLF1 interacting proteins.

Gene Symbol	NPM Nb unique peptides			NPM-MLF1 Nb unique peptides			Complex
	Peak 1	Peak 2	Peak 3	Peak 1	Peak 2	Peak 3	
NPM1	23	25	38	20	22	28	NuRD
CHD4	0	**15**	0	0	**25**	0	NuRD
CHD3	0	**7**	0	0	5	0	NuRD
HDAC2	2	**5**	3	2	**6**	1	NuRD
HDAC1	0	**3**	0	0	**5**	1	NuRD
HDAC3	0	0	0	0	**2**	0	NuRD
MBD3	1	**4**	0	1	1	0	NuRD
MTA2	2	**12**	0	2	**7**	0	NuRD
MTA1	0	**3**	0	0	**5**	0	NuRD
RBBP4	3	3	**4**	3	**5**	3	NuRD
GATAD2A	0	**6**	0	0	**2**	0	NuRD
RBBP7	0	**1**	0	0	0	0	NuRD
PBRM1	**28**	10	0	10	**15**	0	P/BAF
ARID2	**23**	7	0	**12**	10	0	P/BAF
SMARCA2 (BRM)	**17**	12	0	4	**7**	0	P/BAF
SMARCA4 (BRG1)	**14**	**14**	0	3	**10**	0	P/BAF
SMARCC2	**15**	11	0	4	**9**	0	P/BAF
SMARCE1	**6**	3	0	**3**	1	0	P/BAF
SMARCD2	**8**	2	0	0	0	0	P/BAF
SMARCB1	**6**	0	0	1	0	0	P/BAF
SMARCD3	**4**	1	0	0	0	0	P/BAF
SMARCD1	**3**	1	0	0	0	0	P/BAF
ARID1A	0	**1**	0	0	0	0	P/BAF
ARID1B	0	0	0	0	**1**	0	P/BAF
BRD7	**9**	4	0	**4**	3	0	P/BAF
BRD9	0	0	0	**2**	0	**2**	P/BAF
SMARCA5 (SNF2H)	19	**23**	1	12	**23**	6	ISWI
BAZ1B	17	**18**	2	1	**8**	0	ISWI
SMARCA1 (SNF2L)	**12**	9	0	3	**12**	3	ISWI
MYBBP1A	0	0	0	13	**35**	6	ISWI
BPTF	0	0		0	**8**	0	ISWI

Gene symbols, number of unique peptides detected and related remodeling complexes are indicated. The fractions were regrouped according to the size exclusion chromatography graphs presented in Fig 2B. Highlighted numbers are indicating the larger number of peptides detected for each protein (in NPM or NPM-MLF1 samples).

Altogether, the above results suggest that NPM-MLF1 and NPM interact and co-elute with multiple subunits of NuRD, P/BAF and ISWI into complexes of ~2000 kDa and ~1100 kDa in hematopoietic cells. Furthermore, the size fractionation analysis suggests a reorganization of remodeling complexes in NPM-MLF1 expressing cells.

### Identification of wild-type and mutant-NPM-regulated genes: evidence for altered transcriptomes in the etiology of NPM-mutated malignancies

**Fig 3:**
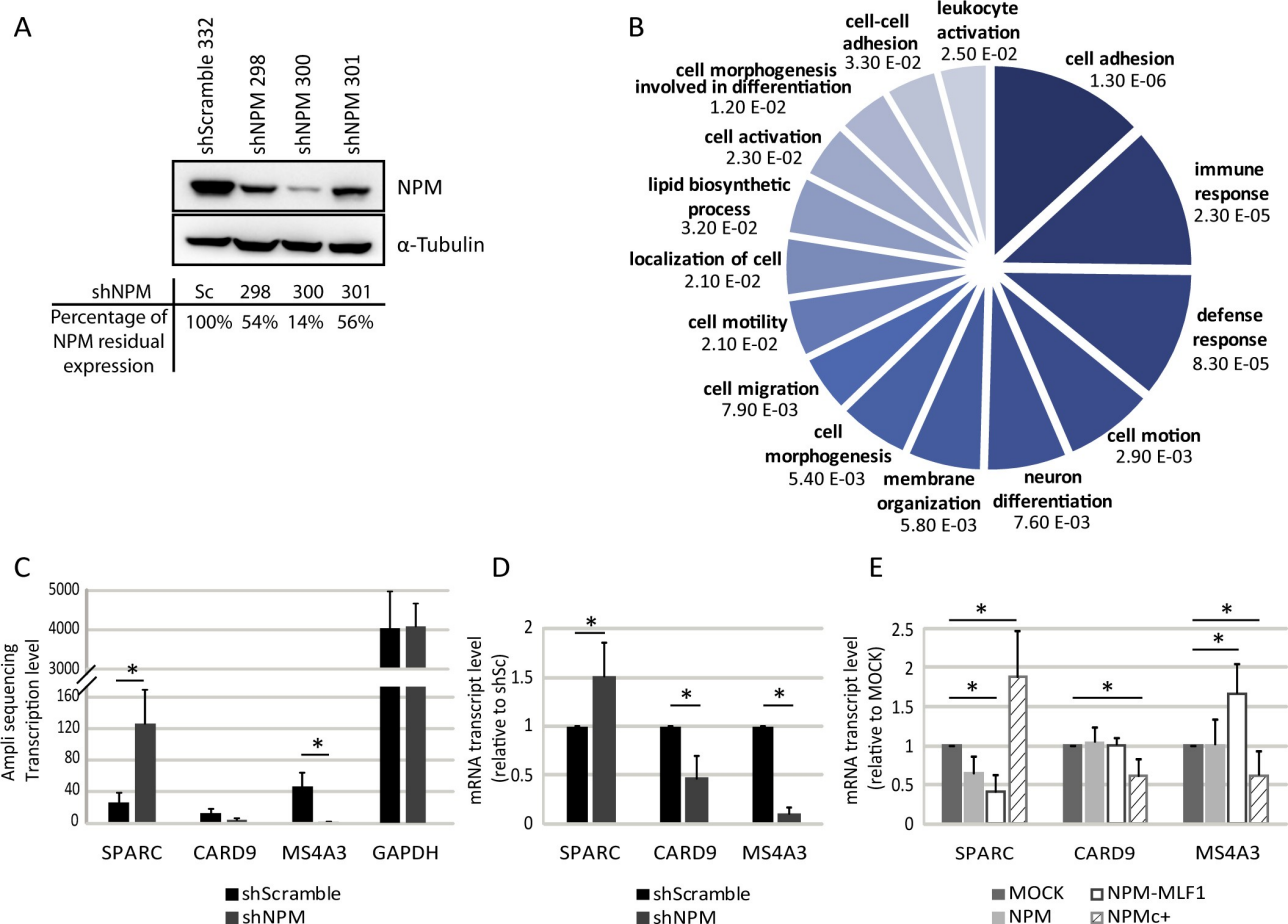
Identification of genes influenced by the knockdown of NPM. **A**) Western blots of whole cell extracts of K562 cells infected with the lentiviral plasmid pLKO.1-puro expressing *NPM1* shRNA (298, 300, 301) or non-target shRNA (Scramble, 332); α–Tubulin was used as loading control; band intensity was calculated with Multi Gauge software; the percentage of residual NPM expression is indicated at the bottom of the panels; **B**) AmpliSeq analysis was performed with total RNA isolated from K562/shScramble (shSc) and K562/shNPM300 (NPM^KD^) cells; genes with significant expression variations (Log2 Fold change ≥ 2 and ≤ -2) were identified and classified according to functional annotation tools on DAVID Bioinformatics Resource v6.8 database; GO Terms corresponding *p*-values are indicated on the pie chart graph; the size of each portion is proportional to the number of genes affected per function; **C-E**) Gene expression; *CARD9*, *MS4A3* and *SPARC* gene expression, such as defined by the AmpliSeq analysis (**C**) or RT-qPCR assays (**D, E**); *GAPDH* was used as internal control; *: *p* ≤0.05 obtained by unpaired Student’s t test.

The expression level of multiple genes is affected in CN-AML patient samples characterized by the NPMc+ mutation [4, 5]. To investigate the effect of NPM nuclear loss-of-function, as in NPMc+ leukemic cells and identify NPM target genes, the knockdown of NPM (NPM^KD^) was performed in K562 cells. Different shRNA were tested and two were selected based on their capacity to reduce NPM levels (shNPM300 and shNPM298; Fig 3A). To identify genes differentially expressed in NPM^KD^, we used AmpliSeq and compared the transcriptome of the shSc cells (scrambled shRNA) with the transcriptome of shNPM300 (NPM^KD^) cells. Based on the correlation plot illustrated in S6A Fig, three NPM^KD^ samples and two shSc samples were retained for comparative analysis. Differentially expressed genes with log2 fold change of ≥ 2 and False Discovery Rate (FDR) of ≤ 0.05 were identified (S6B Fig). Differentially expressed genes with log2 fold change ≥ 1.5 in the NPM^KD^ cells were considered for further analysis (S6C Fig and S4 File). The GO term analysis of these genes, generated with DAVID bioinformatics software, indicated that the genes affected by the NPM^KD^ encode proteins involved in various cellular processes (Fig 3B). Nonetheless, the majority of these genes belong to two processes, namely: (i) cellular adhesion and interaction with the milieu; and (ii) stress related response, including immune response.

To further investigate the transcriptional regulation mechanism(s) influenced by NPM, we selected three genes from the biological processes that were identified (Fig 3B) and affected by NPM^KD^ in K562 cells (S6C Fig). As they are also abnormally regulated in AML samples characterized by NPMc+ mutation [4, 5] we selected: CAspase Recruitment Domain-containing protein 9 (*CARD9*); Secreted Protein Acidic and Rich Cysteine (*SPARC*); and Membrane-Spanning 4-domains subfamily A member 3 (*MS4A3* or *HTm4)*. *CARD9* can activate the NF-κB pathway [31], which is critical to inflammation but also tumor cell survival, migration and proliferation [32]. *CARD9* can be abnormally regulated in NPMc+ CN-AML samples (according to data GEO profiles, NCBI from [5]; accession number: GDS4500) [4]. *SPARC* is downregulated in MDS [33] and upregulated in AML carrying the t(8;12) or inv(16) rearrangement [34] and *SPARC* overexpression promotes Imatinib resistance in chronic myeloid leukemia [35]. SPARC is an extracellular collagen-binding protein affecting cell interactions with their environment [36]. Finally *MS4A3* which is involved in integration of signals from the cell environment [37], was also selected since overexpression of EVI1, a marker of poor prognosis in adult AML, results in *MS4A3* repression [38, 39].

Transcriptome analysis indicated that *CARD9* is downregulated in NPM^KD^ cells (Fig 3C and S4 File). *SPARC* is upregulated in NPM^KD^ cells (Fig 3C; FDR of ≤ 0.05; S3 File), which can also be detected in some NPMc+ CN-AML samples [4, 5]. *MS4A3* is downregulated in NPM^KD^ cells (S4 File and Fig 3C) as well as in NPMc+ CN-AML samples. Transcriptome results for these genes were validated by RT-qPCR in the shNPM300 and shNPM298 (Fig 3D and S7 Fig). In NPMc+ expressing cells, expression of *CARD9*, *MS4A3* and *SPARC* was affected in a similar manner *vs* NPM^KD^ cells (Fig 3C and 3E). However, the relative expression level of *CARD9*, *SPARC* and *MS4A3* was clearly different in NPM-MLF1 expressing cells (Fig 3E). Thus, the effect of NPM-MLF1 on gene regulation differs from the ‘nuclear loss-of-function’ imposed by NPM^KD^ or by expression of NPMc+.

### NPM influences the recruitment of NuRD to target genes

**Fig 4:**
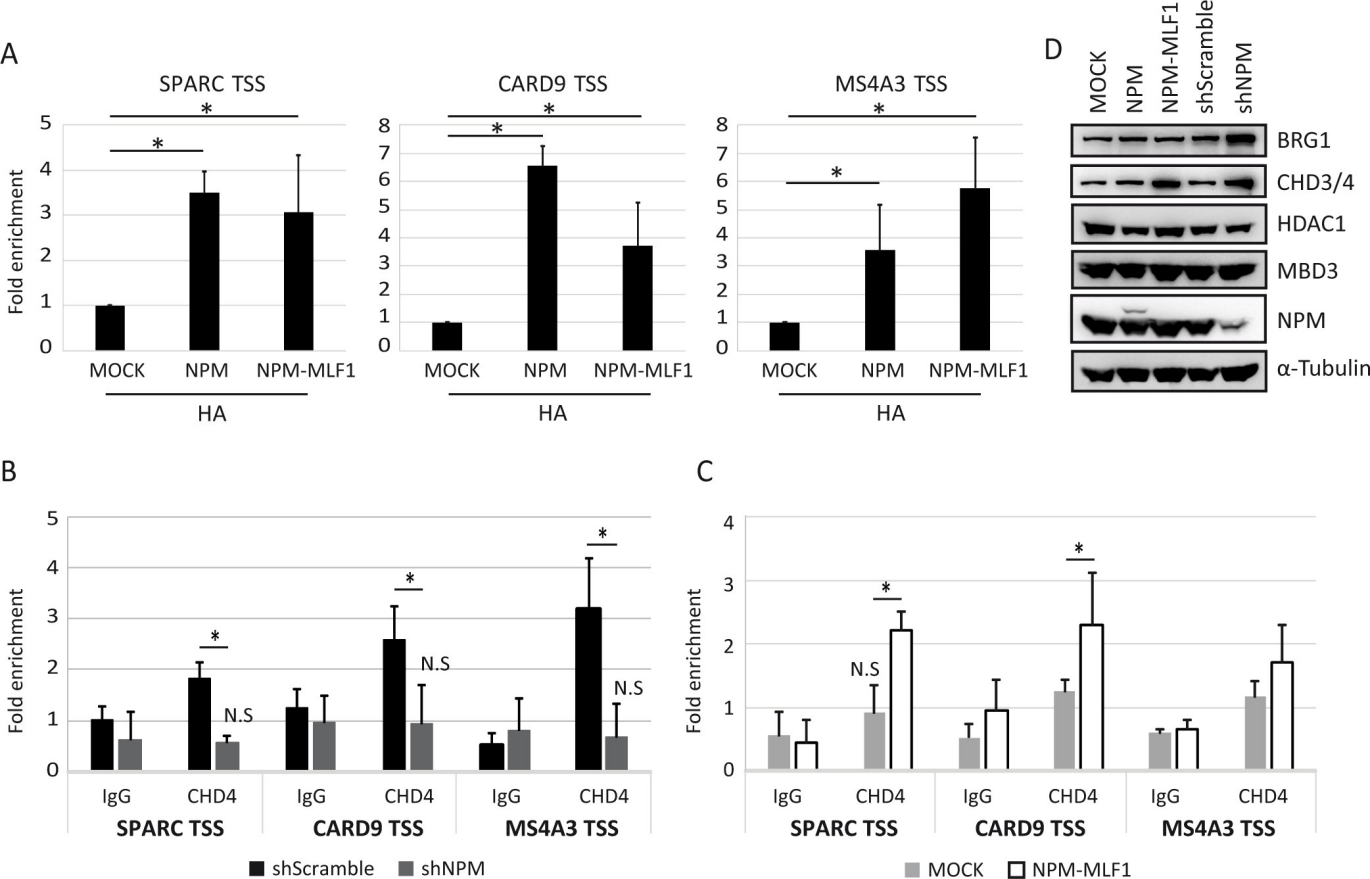
NPM and NPM-MLF1 bind to chromatin and facilitate the recruitment of the CHD4/NuRD complex. **A-C**) ChIP-qPCR assays; chromatin of MOCK, NPM, NPM-MLF1, shScramble (shSc) and ShNPM (shNPM300) cells was immunoprecipitated with HA, CHD4 antibodies or isotype-matched immunoglobulins (IgG); *SPARC* TSS: -120bp to +8bp; *CARD9* TSS: -99bp to -37bp; *MS4A3* TSS: -6bp to +74bp; fold enrichments were relative to the neuronal *Pax6* regulatory element, used as internal control; *: *p* ≤0.05 obtained by unpaired Student’s t test; N.S: Non-significant enrichment compared to corresponding IgG; **D**) Western blots of whole cell extracts; the antibodies used are indicated on the right side of the panels; α-Tubulin was used as loading control.

We first assessed whether FH-NPM or FH-NPM-MLF1 could be recruited to the *SPARC*, *CARD9* and *MS4A3* genes in hematopoietic cells. ChIP-qPCR analysis indicated that FH-NPM and FH-NPM-MLF1 are recruited to the Transcriptional Start Site (TSS) region of *SPARC*, *CARD9* and *MS4A3* (Fig 4A). Next, we questioned whether NPM could influence the recruitment of the NuRD subunit CHD4 to these genes. In shSc cells, CHD4 was detected at the TSS of *SPARC*, *CARD9* and *MS4A3*. CHD4 recruitment was lost in NPM^KD^ cells (Fig 4B). Upon expression of NPM-MLF1, CHD4 significantly increased at *SPARC* and *CARD9* TSS (Fig 4C). Although not significant, changes were also observed at *MS4A3* TSS. Semi-quantitative Western blot analysis revealed higher BRG1 expression in NPM^KD^ cells whereas CHD3/4 levels increased in NPM-MLF1 and NPM^KD^ cells (Fig 4D). Since CHD3/4 upregulation is detectable in both NPM-MLF1 and NPM^KD^ cells, which have an opposite effect on the expression of NPM target genes, it can be assumed that the expression level of CHD3/4 alone is not sufficient to modify the regulation of *SPARC*, *CARD9* and *MS4A3* genes.

Together, these results indicate that NPM and NPM-MLF1 can bind the chromatin of either transcriptionally active or repressed genes. Furthermore, NPM-MLF1 can facilitate the CHD4/NuRD occupancy to genes deregulated. BRG1 recruitment could not be assessed due to very low signal-to-noise ratio. We suspect that the epitope could be masked by surrounding interacting proteins. However, the analysis of ChIP-seq previously performed in K562 cells (GEO accession: GSM1003510 and GSM935633) suggests that CHD4 and BRG1 can both be recruited to the *CARD9*, *MS4A3* and *SPARC* genes (S8 Fig).

### NPM and NPM-MLF1 influence transcription initiation and elongation

**Fig 5:**
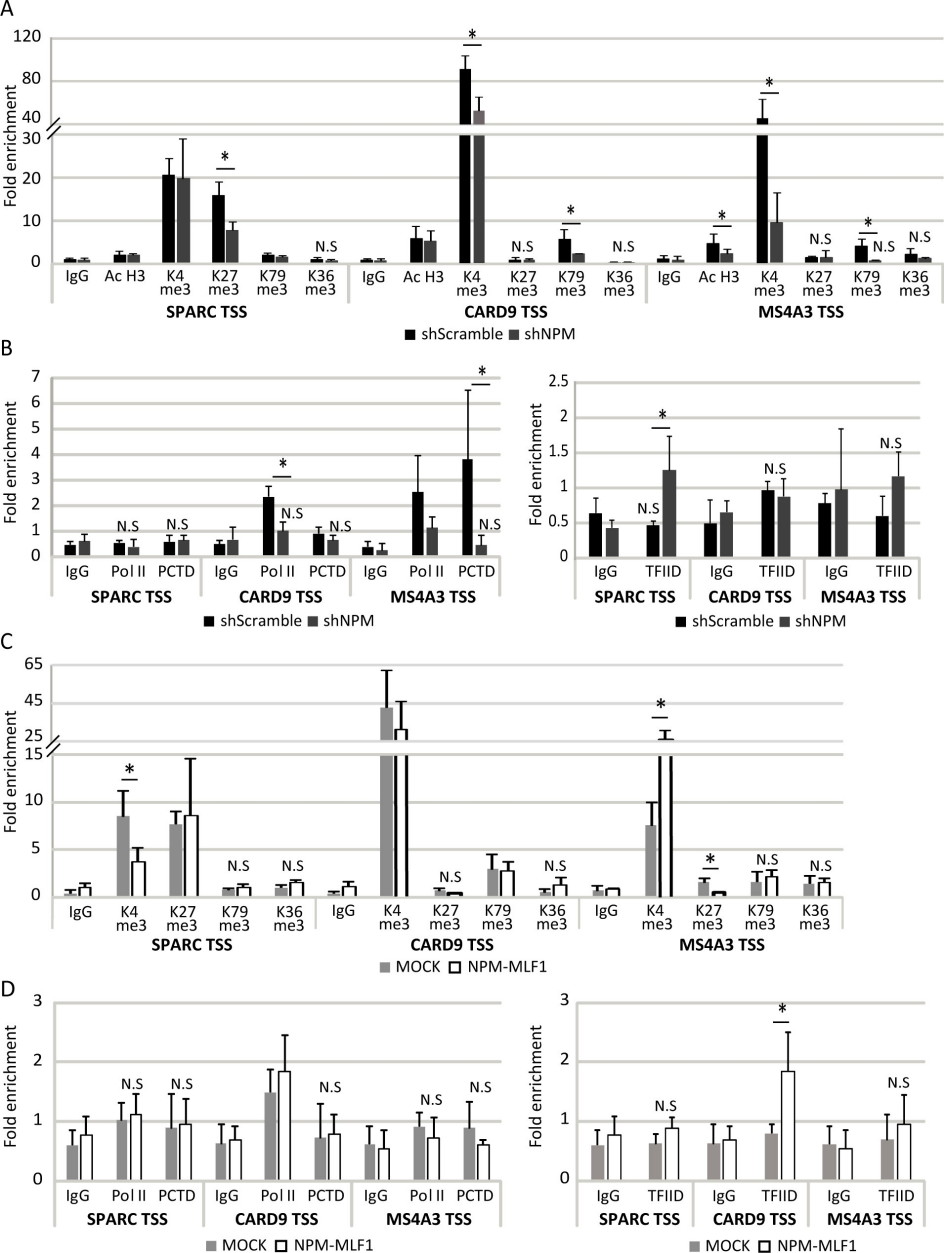
NPM and NPM-MLF1 influence the chromatin organization of *SPARC*, *CARD9* and *MS4A3* genes. **A-D**) ChIP-qPCR assays; chromatin of MOCK, NPM-MLF1, shScramble and shNPM300 (ShNPM) cells was immunoprecipitated with H3ac (K9 and K14 acetylation), H3K4me3, H3K27me3, H3K79me3, H3K36me3, Pol II, Ser2-phosphorylated Pol II C-terminal domain (PCTD), TFIID antibodies or isotype-matched immunoglobulins (IgG); *SPARC* TSS: -120bp to +8bp; *CARD9* TSS: -99bp to -37bp; *MS4A3* TSS: -6bp to +74bp; fold enrichments were relative to the neuronal *Pax6* regulatory element, used as internal control; *: *p* ≤0.05 obtained by unpaired Student’s t test; N.S: Non-significant enrichment compared to corresponding IgG.

We then investigated the effect of NPM and NPM-MLF1 on chromatin and promoter organization as well as transcription elongation at the NPM-activated *CARD9* and *MS4A3* genes and the NPM-repressed *SPARC* gene. Chromatin organization was probed by analysis of histone post-translational modifications by ChIP-qPCR. Specifically, we investigated the enrichment levels of H3K27me3, H3K4me3, H3ac (H3K9ac and H3K14ac), H3K36me3 and H3K79me3 at gene TSS, since (i) NuRD can participate in gene repression with the PRC2 complex [40] and the P/BAF BRG1 can oppose PRC2 activity [20]; and (ii) NuRD and P/BAF regulate gene activation and can participate in transcription elongation [27, 41–43]. *SPARC* TSS region was characterized by H3ac, H3K27me3, H3K4me3 and H3K79me3 marks and only H3K27me3 enrichment levels significantly decreased in NPM^KD^ cells (Fig 5A), suggesting the influence of NPM on the recruitment/activity of the PRC2 enzyme EZH2 to this gene. The effect of NPM^KD^ was the same in the shNPM298 cells (S9 Fig). The influence of NPM on PRC2 recruitment is supported by the co-IPs of NPM with the PRC2 subunits EZH2 and SUZ12 (S10 Fig). The enrichment of both H3K27me3 and H3K4me3 at *SPARC* in shSc cells is consistent with the notion of a bivalent/poised chromatin organization favored by NPM recruitment [44–46]. However, the investigation of promoter organization suggests that NPM-NuRD might also preclude efficient transcription pre-initiation complex (PIC) formation at *SPARC* gene as evidenced by the absence of transcription proficient Ser2-phosphorylated Pol II C-terminal domain (PCTD) and the enrichment of TFIID at *SPARC* TSS only in NPM^KD^ cells (Fig 5B). Rather H3ac, H3K4me3 and H3K79me3 marks, characterized the chromatin of *CARD9* and *MS4A3* TSS but no significant H3K27me3 enrichment could be detected (Fig 5A). H3K4me3 and H3K79me3 characterize transcriptionally active genes where productive transcription elongation is associated with activity of the histone methyltransferases MLL/SETD1 and DOT1L [44]. In NPM^KD^ cells, *CARD9* and *MS4A3* downregulation was accompanied by a significant decrease of H3K4me3 and H3K79me3 levels (Fig 5A), suggesting that NPM recruitment favors *CARD9* and *MS4A3* productive transcription elongation. The possibility that NPM could have a positive effect on *CARD9* and *MS4A3* transcription elongation was also supported by: (i) the significant decrease of PCTD levels at *CARD9* ORF and *MS4A3* TSS in NPM^KD^ *vs* shSc cells (Fig 5B and S11 Fig); and (ii) the interaction of NPM and NPM-MLF1 with the transcription super-elongation complex subunit RBBP5 [47] and the P-TEFb subunit CDK9 (S12 Fig), which is responsible for Pol II Ser2 phosphorylation and, thereby, for the release of promoter-proximal paused Pol II [48]. As observed in the shSc cells, also in MOCK cells the *SPARC* TSS was characterized by H3K4me3 and H3K27me3 marks; moreover, H3K4me3 enrichment significantly decreased in NPM-MLF1 expressing cells (Fig 5C). Consistent with RT-qPCR expression data (Fig 3E), no significant difference was noted in chromatin organization of *CARD9* TSS between MOCK and NPM-MLF1 expressing cells (Fig 5C). However, in MOCK cells, H3K4me3 and H3K27me3 marked the *MS4A3* TSS, and the H3K4me3 enrichment significantly increased whereas H3K27me3 enrichment significantly decreased in NPM-MLF1 expressing cells (Fig 5C). According to TFIID, Pol II and PCTD recruitment, NPM-MLF1 has no significant effect on the organization of the transcription PIC of *SPARC*, *CARD9* or *MS4A3* (Fig 5D). Indeed, the significant increase of TFIID recruitment to *CARD9* is non consistent with a modulation of PIC formation since no variation of Pol II or PCTD could be detected to *CARD9*.

In conclusion, NPM-NuRD and NPM-MLF1-NuRD can participate in both, transcription repression as well as activation. In its role as an activator, the NPM-NuRD complex is likely to be linked to productive transcription elongation, as observed at the transcriptionally active *CARD9* and *MS4A3* genes. As transcriptional repressor at the *SPARC* gene, the NPM-NuRD complex can promote bivalent/poised chromatin organization characterized by H3K27me3 and H3K4me3 [45, 46]. The NPM-MLF1 recruitment to *SPARC* enhances NuRD recruitment, which is associated to the disruption of the bivalent/poised chromatin organization and to further repression of this gene.

## Discussion

We report that NPM and NPM-MLF1 interact with subunits of the chromatin remodeling complexes NuRD, P/BAF, and with complexes of the ISWI family. The proteomics analysis suggests that NPM-MLF1 influences the organization of the remodeling complexes NuRD and P/BAF in hematopoietic cells. Additionally, a direct implication of NPM in gene regulation is demonstrated by (i) NPM recruitment to specific genes concomitant with the binding of NuRD; and (ii) the uncontrolled recruitment of the CHD4/NuRD complex in NPM-deficient or NPM-MLF1-expressing cells. We demonstrate that the non-nucleolar accumulation of NPM-MLF1 directly influences gene expression and can affect recruitment of the remodeling complex NuRD to NPM regulated genes. In contrast, the effect of NPMc+ is similar to that of NPM^KD^ and, hence, related to a nuclear loss-of-function.

The cytoplasmic accumulation of NPMc+ promotes leukemia [24]. Like NPMc+, NPM-MLF1 maintains the capacity to interact with and induces the cytoplasmic relocalization of the non-mutated NPM. This is believed to result in a loss of NPM nuclear function and trigger leukemia development [15, 16]. This view is supported by the previous demonstration that inhibition of interaction between NPM and NPMc+ favors hematopoietic cell differentiation and loss of AML cell survival [49]. To elucidate the nuclear functions of NPM and define the effect of NPM-MLF1, we identified proteins interacting with NPM and NPM-MLF1. Protein interactome analysis led to the identification of new NPM and NPM-MLF1 partners, which belong to the remodeling complexes NuRD, P/BAF and complexes of the ISWI family. They are known organizer of the chromatin architecture. We focused on the interactions with NuRD and P/BAF since the MS analysis suggested that their subunits preferentially interact with the oncoprotein NPM-MLF1 (*vs* NPM; Table 1) and hence, could explain gene deregulation associated with NPM-MLF1 expression. This preferential interaction was not observed with SNF2H and SNF2L, the nucleosome remodeling ATPases alternatively included in the ISWI complexes [22].

The literature is relatively silent with respect to any role(s) for NPM in chromatin structure/remodeling. Analysis of the NPM protein interactome in hepatoma cells [50] as well as a high-throughput proteome analysis made in HeLa cells [51] indicated that in addition to interactions with proteins affecting various cellular mechanisms, NPM interacts with histones and a few factors involved in gene regulation. The high-throughput analysis made in HeLa cells identified the ISWI complex subunit SNF2H as a potential interacting partner of NPM [51]. As many as 14 different ISWI complexes have been described [22]. Our protein interactome and co-IP results demonstrate that in fact, NPM and NPM-MLF1 can interact with different subunits (SNF2H, SNF2L and BAZ1B/WSTF; Fig 1D) specific to distinct ISWI complexes. Whether NPM and NPM-MLF1 can be recruited to chromatin with different ISWI complexes remains unknown. However, the unique composition of different ISWI complexes could be required for the participation of NPM (and NPM-MLF1) to several nuclear mechanisms such as transcriptional regulation, DNA repair, and DNA replication.

The interaction of NPM with BRG1 was reported in APL cells [8] where it was demonstrated that the translocation product PML-RARA favors the formation of an atypical transcriptional repressive complex composed of NPM/TOP2B/BRG1 [8]. Our proteomic results indicate that NPM also interacts with BRG1 in hematopoietic cells that are not characterized by the PML-RARA translocation. Interestingly, P/BAF can recruit TOP2A and TOP2B enzymes (both identified in the MS analysis of the NPM-MLF1 interactome; S1 File) to facilitate DNA decatenation and heterochromatin remodeling [52, 53]. Thus, NPM and NPM-MLF1 might be associated with TOP2 enzymes when interacting with P/BAF.

Our results on the effects of NPM^KD^ and NPM-MLF1 expression prompted us to identify genes abnormally regulated under these conditions. Specifically, we found that NPM and NPM-MLF1 could bind the chromatin of deregulated genes and influence the recruitment of CHD4/NuRD. Their targeting to genes is most likely mediated by specific transcription factors and some of them, *e*.*g*. YY1 and CTCF, were identified by MS interactome analysis (S2 File). However, when directly assessed by protein co-IP, we observed that both NPM and NPM-MLF interact similarly with YY1 and CTCF (S13 Fig). It remains to be defined whether other transcription factors or DNA structures [54] could differentially interact with NPM *vs* NPM-MLF1 and induce the preferential nuclear localization of NPM-MLF1 over NPM. Nonetheless, the enhanced recruitment of CHD4/NuRD imposed by NPM-MLF1 is likely to be critical for the leukemogenic activity of this fusion oncoprotein. The influence of NPM and NPM-MLF1 on P/BAF recruitment was not directly tested, but the comparative analysis of published ChIP-seq results obtained in K562 cells (S8 Fig), suggests that CHD4 and BRG1 can both be recruited to the NPM-activated genes *CARD9* and *MS4A3*, as well as to the NPM-repressed gene SPARC. Whether CHD4 and BRG1 could be simultaneously recruited to these NPM regulated genes could not be clarified. Indeed, the proteomic analysis performed was not conclusive since, the size-exclusion chromatography suggests that when interacting with NPM (or NPM-MLF1), CHD4 and BRG1 could be in the same complex and elute in same fractions of size exclusion chromatography, but CHD4 and BGR1 co-immunoprecipitation could not be detected in the K562 cells.

The recruitment of CHD4 and BRG1 to the same genes has been reported by different groups. Indeed, these nucleosome remodeling activities were reported to be associated and involved in the repression of γ-genes during β-globin gene switching [55, 56]. Alternatively, the recruitment of NuRD and BAF complexes to the same genes was reported during epithelial-mesenchymal transition in oral cancer cells [57]. Then, NuRD was found to antagonize BAF complex activity during the transition from epithelial to mesenchymal, suggesting that the recruitment of NuRD and BAF to common genes could facilitate the switching between transcriptional activation and repression. NuRD and BAF also show similar chromatin binding in embryonic stem cells [55, 58, 59]. Thus, such as proposed in embryonic stem cells [60], these examples suggest that the potential opposing activity of CHD4/NuRD and BRG1/BAF could facilitate the fine-tuning of gene expression and/or their capacity to respond to activation or repression signals [58].

NuRD is a critical regulator of chromatin organization within gene regulatory regions and, thereby, can facilitate different events leading to transcription initiation. Importantly, as demonstrated for certain ISWI complexes [60, 61], NuRD also influences transcription elongation [27, 41]. One of our goals was to understand how NPM and NPM-MLF1 act with NuRD to promote transcriptional activation or repression. Our results indicate that NPM-NuRD is required for the transcriptional repression of *SPARC* and activation of *CARD9* and *MS4A3*. In the case of *SPARC*, NPM-MLF1 favors NuRD recruitment with consequent decrease of gene expression. Enhanced recruitment of TFIID at the TSS in NPM^KD^ cells suggests that NPM-NuRD negatively regulates PIC formation at *SPARC*. Additionally, difference in H3K27 methylation suggests that when implicated in gene repression, NPM-NuRD (or NPM-MLF1-NuRD) act with the PRC2 methyltransferase EZH2. The chromatin and PIC analyses suggest that NPM-NuRD favors a bivalent chromatin characterized by H3K4me3 and H3K27me3, and the NPM-MLF1 recruitment disrupts this chromatin organization to further repress *SPARC* transcription. Thus, NPM and NPM-MLF1 regulate *SPARC* transcription by controlling the chromatin organization and consequently, transcriptional activation.

With respect to *CARD9* and *MS4A3*, NPM^KD^ does not influence TFIID recruitment but significantly affects H3K4me3, H3K79me3 and PCTD enrichment, suggesting that transcription elongation but not PIC formation or transcription initiation would be impaired by NPM^KD^ [27]. Indeed, H3K4me3 and H3K79me3 are formed by the histone methyltransferases MLL/SETD1 and DOT1L, respectively [62], and these chromatin marks can be associated with the transcription elongation complex thus promoting productive transcription elongation. The elongation complex including the MLL/SETD1 and DOT1L activities also contains P-TEFb [47, 63] and NPM was reported to interact with HEXIM, a subunit of the repressed form of P-TEFb [64]. The NPM-HEXIM interaction was not detected in K562 cells (LC-MS/MS analysis; S1 File). However, NuRD and P-TEFb are found in a larger complex that facilitates transcription elongation [27, 41] and NPM as well as NPM-MLF1 co-immunoprecipitate with the P-TEFb catalytic subunit CDK9 (S12 Fig). Thus, the positive influence of NPM-NuRD on transcription elongation of *CARD9* and *MS4A3* suggests that NPM-NuRD could act with P-TEFb at these genes. The absence of variation of *CARD9* expression in MOCK and NPM-MLF1 expressing cells could suggest that NuRD recruitment to *CARD9* is optimal under normal conditions, and the enhanced recruitment of NuRD in NPM-MLF1 expressing cells would not further promote transcription elongation at this gene. Importantly, *CARD9* is a NF-κB inducible gene [31] and may require the binding of this stress-induced transcription factor to be further activated. Thus, the NPM-NuRD effect on *CARD9* and *MS4A3* transcription is possibly related to improvement in transcription elongation since NPM interacts with CDK9/P-TEFb, and it favors MLL/SETD1 and DOT1L activities. It is not clear at present whether NPM-MLF1-NuRD can similarly affect transcription or acts more specifically on the chromatin organization at promoters.

In summary, the mutation of subunits of ATP-dependent chromatin remodeling complexes can favor leukemia [65–67]. Our study provides evidence that NPM and the leukemogenic translocation NPM-MLF1 interact with the chromatin remodeling complexes NuRD, P/BAF as well as complexes of the ISWI family. We show that NPM and NPM-MLF1 can both recruit and promote the capacity of the CHD4/NuRD complex to regulate gene expression in hematopoietic cells. NPM nuclear deficiency results in the abnormal regulation of NPM- and CHD4/NuRD-target genes. The oncoprotein NPM-MLF1 maintains the capacity to interact with NPM and is partially located in the cytoplasm. Nonetheless, unlike the nuclear ‘loss-of-function’ imposed by NPM^KD^ or NPMc+, the nuclear NPM-MLF1 binds to the chromatin, influences the organization of remodeling complexes, favors the uncontrolled recruitment of NuRD and induces abnormal transcriptional regulation of NPM target genes. The balance between remodeling complex NuRD and BAF is critical for the regulation of genes [57]. We propose that NPM-MLF1 disrupts the balance between NuRD and P/BAF complex to certain genes and thereby, induces their abnormal regulation. The results presented here suggests that NPM-MLF1 is associated to nuclear gain-of-function, rather than by a previously proposed loss of nuclear function effect, such as imposed by NPM^KD^ or NPMc+. The discovery of new protein partners of NPM and NPM-MLF1 and demonstration of mechanisms used by these factors to regulate genes provide a new basis for our understanding and further investigation of the NPM-related oncoprotein driven AML and their poor prognosis, especially those carrying the oncogenic fusion NPM-MLF1.

## Materials and methods

### Cell culture and stable cell transfections

The human chronic myeloid leukemia blast crisis cell line K562 was purchased from the American Type Culture Collection (Rockville, MD, USA) and cultured in RPMI-1640 medium supplemented with 10% fetal bovine serum at 37°C. K562/MOCK, K562/NPM, K562/NPM-MLF1 and K562/NPMc+ (hereafter: MOCK, NPM, NPM-MLF1 and NPMc+) cells were obtained by electroporation with linearized pOZN-Flag-HA-IRES-IL2R (pOZN) empty vector or pOZN-NPM, pOZN-NPM-MLF1, or pOZN-NPMc+ vectors. The selection of positive clones stably expressing NPM or mutants was conducted using anti-IL-2 coated magnetic beads was performed as previously reported [68]. NPM knockdown or scramble K562 clones (respectively: NPM^KD^ and shSc) were obtained by lentiviral infection with pLKO.1-puro plasmid vectors (Sigma). Stable clones were selected with 2 μg/mL of Puromycin.

### Nuclear extraction and co-immunoprecipitation assays

Nuclear extracts were prepared as previously described [68]. Briefly, cells were incubated for 10 min on ice in a hypotonic buffer (10 mM Tris pH 7.3, 10 mM KCl, 1.5 mM MgCl_2_). Cells were then lysed using a dounce homogenizer and isolation of nuclei was monitored under the microscope. After centrifugation, the nuclear pellet was resuspended in two equivalent volume of low salt buffer (20 mM Tris pH 7.3, 25% Glycerol, 0.2 mM EDTA, 20 mM KCl, 1.5 mM MgCl_2_). One volume of high salt buffer (20 mM Tris pH 7.3, 25% Glycerol, 0.2 mM EDTA, 1.2 M KCl, 1.5 mM MgCl_2_) was then added dropwise in the cold room. Following extraction of nuclear protein, chromatin was separated by ultracentrifugation (15000 rpm, 30 minutes and then 20000 rpm, 20 minutes at 4°C). All buffers were completed with 1% protease inhibitor cocktail (Bimake, Catalog Number: B14001), 1 mM PMSF, 10 mM β mercapto-ethanol. For Co-immunoprecipitations (co-IP), the nuclear extract was centrifuged at 13000 rpm for 10 minutes at 4°C and the supernatants were diluted in the equivalent volume of IP buffer (50 mM Tris pH 7.5, 10% Glycerol, 0.2 mM EDTA, 145 mM KCl, 5 mM MgCl_2_, 0.5% NP40, 1% protease inhibitor cocktail (Bimake), 1 mM PMSF, 10 mM β mercapto-ethanol). Specific antibodies or isotype-matched immunoglobulin controls (IgG) (2 μg) were added and lysates were incubated overnight at 4°C. Complexes were precipitated with protein G-Sepharose beads (P3296, Sigma) or Flag agarose beads (A2220, Sigma) and washed three to six times with 1 ml of ice-cold IP buffer. The antibodies used are listed in S1 Table.

### Sequential immunoaffinity purification (Tap-Tag) and Fast protein liquid chromatography (FPLC)

Flag-HA sequential immunoaffinity purification was carried out as previously described [68], from 20 mg of nuclear lysates of K562 (MOCK, NPM and NPM-MLF1) cells. Immunoprecipitated proteins were eluted from Flag or HA beads with 4 μg/ml of Flag or HA polypeptides (sequences: Flag: DYKDDDDK or HA:YPYDVPDYA from BioBasic) and molecular size chromatography was performed on the immune-affinity purified complexes with the AKTA purifier FPLC system (GE Healthcare) using Superose 6 increase 10/300 GL column (GE Healthcare). The column was calibrated with Dextran (2000 kDa), Thyroglobulin (669 kDa), Aldolase (158 kDa) and Bovine Serum Albumin (67 kDa) in FPLC buffer (50 mM Tris pH 7.3, 10% Glycerol, 60 mM KCl, 0.2 mM EDTA pH 8.5 mM MgCl_2_, 1% NP40 and 0.5 mM DTT). 500 μL of nuclear lysate or Tap-Tag purified sample was loaded on the column. After 0.2 column volume of isocratic elution, 0.5 mL fractions were collected, and proteins were precipitated with 15% TCA. Immunopurified samples or fractions, were first analyzed by silver staining, and then LC-MS/MS analysis was performed to identify interacting partners (Taplin Mass Spectrometry Facility, Harvard University, Boston).

Western blots were performed according to standard protocols. The antibodies used are listed in the S1 Table. Images were acquired with Image Quant LAS400 device (GE Healthcare Life Sciences).

### Chromatin immunoprecipitation and quantitative PCR analyses

Chromatin immunoprecipitation (ChIP) and quantitative real-time PCR assay were performed as previously described [56]. Briefly, 1 x 10^6^ cells were fixed with 1.5 mM ethylene glycolbis[succnimidyl succinate] (EGS; MJS Biolynx Inc), 1% formaldehyde (Sigma) and quenched with 50 mM Glycine (Sigma). The antibodies used are indicated in S1 Table. About 10% of immunoprecipitated material and 3% of input sample were used as template for qPCR with SYBR green (Bimake) on a 7500 Real time PCR System (Applied Biosystems). Primer sequences are described in S2 Table.

### Quantitative reverse transcription PCR (RT-qPCR)

RNA was extracted with TRIzol (Invitrogen) and treated with DNaseI-RNase-free (Thermo Scientific). Reverse transcription reactions were performed with oligo(dT)_18_ primers (Thermo Scientific) and RevertAid reverse transcriptase (Thermo Scientific), according to manufacturer’s instructions. RT material (1/20) was used as template for qPCR with SYBR green (Biotool) on a 7500 Real time PCR System (Applied Biosystems). Primer sequences are described in S2 Table. Quantification was carried out as previously described [56].

### Immunofluorescence

5 x 10^5^ cells were cytospun on slides and fixed with 3% paraformaldehyde for 30 minutes. Slides were washed with immunofluorescence (IF) wash buffer (0.1% Triton, 1 mM Sodium Azide in PBS) and blocked with 5% Bovine Serum Albumin for 1 hour. Cells were first stained with rabbit anti-HA and mouse anti-NPM and then with Alexa Fluor 594-conjugated anti-rabbit and Alexa Fluor 488-conjugated anti-mouse antibodies (S1 Table). Slides were mounted with DAPI-Dabco:Vectashield solution (1:1). Images were acquired with Delta Vision Core/Olympus IX71 microscope and were deconvoluted at 200 nm with SoftWoRx 6.5.2 software.

### Cell cycle analysis

2 x 10^6^ cells were washed with cold PBS, fixed with 75% cold ethanol and incubated overnight at -20°C. Cells were then washed with PBS and treated with 100 μg/mL RNase A (Sigma) for 20 minutes at 37°C. DNA was stained with 50 μg/mL Propidium Iodide. Cell cycle analysis was carried out on a FACScan flow cytometer with CellQuestPro software (BD Biosciences) according to the DNA content [69].

### Subcellular fractionation

Subcellular fractionation was performed as previously described [70]. Briefly, cells were lysed for 10 minutes on ice with 0.2 ml hypotonic buffer (10 mM HEPES pH 7.6, 10 mM KCl, 1.5 mM MgCl_2_, 0.5% NP40) supplemented with 1% protease inhibitor cocktail and phosphatase inhibitors (Bimake). After centrifugation, the cytosolic fraction was collected, whereas the nuclear pellet was resuspended in 0.1 mL CSK buffer (20 mM HEPES pH 7.6, 100 mM NaCl, 300 mM Sucrose, 3 mM MgCl_2_, 1 mM CaCl_2_, 0.5% Triton) supplemented with 1% protease inhibitor cocktail (Bimake) and incubated for 3 minutes on ice. Samples were centrifuged and supernatants corresponding to the nucleoplasmic fraction were obtained. Chromatin pellets were resuspended in CSK buffer supplemented with 30 U of Micrococcal nuclease (MNaseI; BioShop) and incubated 30 minutes at room temperature. Chromatin bound proteins were released from DNA with 200 mM ammonium sulfate.

### Ampli-sequencing assay

cDNA libraries were prepared from 10ng of K562 mRNA pre-treated with DNaseI, in triplicate, with the Ion AmpliSeqTM Transcriptome Human Gene Expression Kit (Thermo Fisher Scientific) according to manufacturer's instructions. Briefly, mRNA were reverse transcribed using SuperScript VILO cDNA synthesis kit (Thermo Fisher Scientific) and amplified with Ion AmpliSeq HIFI Mix. Primer sequences were partially digested with FuPa Reagent, and then barcoded using Ion Xpress Barcodes (Thermo Fisher Scientific). Purification was carried out by AMPure XP Reagent (Beckman Coulter). Libraries concentrations were defined by qPCR using Ion Library Quantification kit (Thermo Fisher Scientific). Libraries were pooled together at 50 pM for emulsion PCR, carried out using the Ion Chef Instrument (Thermo Fisher Scientific). Purified Ion Sphere Particles were loaded on Ion P1 Chip. Loading density was around 80%. The sequencing was performed on Ion Proton system (Thermo Fisher Scientific). Ion Torrent software, Torrent Suite v4.4 (Thermo Fisher Scientific) was used for base calling, alignment to the human reference genome (hg19) and quality control. Raw reads were then analyzed automatically using the AmpliSeqRNA plugin to generate gene-level expression values for all 20802 RefSeq human genes.

### Statistical analysis

Unpaired Student's *t*-test was used to determine the level of statistical significance (*P*-value).

## Supporting information

S1 Table List of antibodies. (DOCX)

S2 Table Primer sequence table. (DOCX)

S1 Fig Characterization of the MOCK, NPM, NPM-MLF1 and NPMc+ cell models. **A)** Schematic representation of NPM, NPM-MLF1 and NPMc+ protein structures. The nuclear export signals (NES), nuclear localization signals (NLS) and nucleolar localization signal (NoLS) are indicated; amino acid numbers are specified under the schematic representation of each proteins; the red star represents the NPMc+ mutation. **B**) Western blots of whole cell extracts of K562 cells infected with the pOZN empty vector (MOCK), FH-NPM (NPM), FH-NPM-MLF1 (NPM-MLF1) or FH-NPMc+ (NPMc+) cDNA; the antibodies used are indicated on the right side of the panels; α-Tubulin was used as loading control; molecular weights are indicated on the left side of the panels; **C**) Gene expression; the level of endogenous or transgenic *NPM* was obtained by RT-qPCR; the expression levels were normalized to MOCK cells and *GAPDH* was used as internal control; **D**) Representative cell cycle results; cells were stained with PI and analyzed by FACS; the bar graphs indicate the percentage of cells in each phase of the cycle, apoptosis and polyploid fractions; **E)** Growth curves of MOCK, NPM, NPM-MLF1 and NPMc+ cells. On the first day (D0), cell concentrations were adjusted to 100 000 cells/mL. Cells were counted every 24h for 4 days (D1 to D4); **F,G,I)** Representative immunofluorescence images; (Panel F) HA-Flag proteins were stained with rabbit anti-HA and Alexa fluor 594-conjugated anti-rabbit antibodies (red signals); (Panel G-I) anti-NPM antibodies to detect the endogenous NPM protein (in K562; S1I Fig) or NPMc+ (in OCI-AML3; S1G Fig) leukemia cells; nuclei were counterstained with DAPI (blue signals); the cell model is indicated on the top of the panels. **H)** Cytoplasm, nucleoplasm and chromatin compartments were separated by cell fractionation from MOCK, NPM, NPM-MLF1 and NPMc+ cells; NPM was detected by Western blot; α-Tubulin, APE1 and Histone H3 were used as controls of the different fractions.
(TIF)

S2 Fig Functional grouping and classification of proteins interacting with NPM and NPM-MLF1. Analysis of LC-MS/MS data performed with STRING database. NPM and NPM-MLF1 interacting proteins are classified according to biological processes.
(TIF)

S3 Fig Quality control of the samples. DNA detection on agarore gel loaded with 10μg of K562 whole cell extract lysate, and K562 nuclear extract treated or not with 10μg/mL of DNaseI.
(TIF)

S4 Fig Size exclusion chromatography fractionation of nuclear extracts. Size-exclusion chromatography (FPLC) from K562 nuclear extracts of NPM and NPM-MLF1 expressing cells, fractionated on Superose 6 10/300GL column; the antibodies used for Western blot are indicated on the right side of the panels; average fraction size in KDa is indicated under the panels; NE: nuclear extract; #: lower band (background) corresponding to MBD3 since the NPM detection was performed on membranes previously used for MBD3 detection.
(TIF)

S5 Fig FPLC column calibration and CHD4, BRG1 co-immunoprecipitations. **A)** Calibration of the Superose 6 increase 10/300 GL column; molecular weight fractionation of Dextran (2000 kDa; fractions 7–8), Thyroglobulin (669 kDa; fraction 17), Aldolase (158 kDa; fraction 22) and Bovine serum albumin (67 kDa; fractions 24–25); **B)** Co-Immunoprecipitation with CHD4 and BRG1 antibodies of MOCK, NPM and NPM-MLF1 nuclear extracts; IgG: corresponding isotype-matched immunoglobulins; proteins detected by Western blot are indicated on the right side of the panels.
(TIF)

S6 Fig Transcriptome analysis in NPM knockdown cells. **A)** Correlation plot corresponding to samples used for the transcriptome analysis. Samples 1–3 are triplicates of shScramble samples; samples 4–6 are triplicates of shNPM300 samples; the correlation coefficient r is indicated; **B)** List of genes obtained from the transcriptome analysis that are characterized by a Log2 Fold change expression ≥2 and a fold discovery rates of ≤0.05; *p-values* and Log2 Fold changes are indicated in the table; **C)** List of overlapping genes obtained by microarray analysis from NPMc+ AML patients (Verhaak, *et al*., Blood, 2005) (4) and the AmpliSeq analysis performed on K562 cells; genes with a fold change expression ≥ 1.5 in shNPM vs shScramble cells (AmpliSeq) were retained; upregulated genes are depicted in red, downregulated genes are depicted in blue.
(TIF)

S7 Fig Expression of *CARD9*, *SPARC* and *MS4A3* genes with shNPM298. RT-qPCR assays; *CARD9*, *MS4A3* and *SPARC* gene expression; the expression levels were normalized to shSc cells and *GAPDH* was used as internal control; *: p ≤0.05 obtained by unpaired Student’s t test.
(TIF)

S8 Fig ChIP Sequencing analysis. Analysis of CHD4 and BRG1 enrichment on *SPARC* TSS, *CARD9* TSS and *MS4A3* TSS. *LINGO2* is presented as a negative control region, showing no enrichment for CHD4 or BRG1 and Human Beta globin (HBB) as positive control; chromosomal positions are depicted on the top of each panel; enrichment scale is depicted on the left side of the panels. Importantly, the background level has been substracted and hence, the amplification peaks present regions enriched above the background level; ChIP-Seq data were obtained from K562 cells (ENCODE encyclopedia); BRG1 ChIP-Seq data accession number: GSM935633 and CHD4 ChIP-Seq data accession number: GSM1003510. Data were analysed with the Integrative Genomics Viewer (IGV) software (Robinson, *et al*., Nature biotechnology, 2011 and Thorvaldsdottir, *et al*., Briefings in Bioinformatics, 2013) and mapped on the hg19 genome.
(TIF)

S9 Fig Supplementary data for histone modification enrichments to *SPARC* and *CARD9* TSS. ChIP-qPCR assays; chromatin of shScramble (shSc) and ShNPM298 cells were immunoprecipitated with H3K4me3, H3K27me3, H3K79me3 and H3K36me3 antibodies or isotype-matched immunoglobulins (IgG); *SPARC* TSS: -120bp to +8bp; *CARD9* TSS: -99bp to -37bp; fold enrichments were relative to the neuronal *Pax6* regulatory element, used as internal control; *: p ≤0.05 obtained by unpaired Student’s t test; N.S: Non-significant enrichment compared to corresponding IgG.
(TIF)

S10 Fig NPM interacts with the PRC2 complex. The co-immunoprecipitations were performed with EZH2 or Flag antibodies on MOCK and NPM nuclear extracts; IgG: corresponding isotype-matched immunoglobulins; The proteins detected by Western blot are indicated on the right side of the panels.
(TIF)

S11 Fig Supplementary data for Pol II and PCTD enrichment in the ORF of *SPARC* and *CARD9*. ChIP-qPCR assays; chromatin of shScramble, shNPM300 (ShNPM), MOCK, NPM-MLF1 cells was immunoprecipitated with Pol II, Ser2-phosphorylated Pol II C-terminal domain (PCTD) or isotype-matched immunoglobulins (IgG); *SPARC* ORF: +24165bp to +24278bp; *CARD9* ORF: +6132bp to +6214bp; fold enrichments are relative to the neuronal *Pax6* regulatory element, used as internal control; *: p ≤0.05 obtained by unpaired Student’s t test; N.S: Non-significant enrichment compared to corresponding IgG.
(TIF)

S12 Fig NPM-MLF1 co-immunoprecipitations with P-TEFb and Super elongation complex. Co-immunoprecipitation experiments were performed with MOCK, NPM or NPM-MLF1 cell nuclear extracts, and with Flag, CDK9 antibodies or isotype-matched immunoglobulins (IgG); the proteins detected by Western blot are indicated on right side of the panels.
(TIF)

S13 Fig NPM and NPM-MLF1 interact with CTCF and YY1 transcription factors. The co-immunoprecipitations were performed on MOCK, NPM or NPM-MLF1 cell nuclear extracts with Flag, CTCF, YY1 antibodies or isotype-matched immunoglobulins (IgG); the proteins detected by Western blot are indicated on right side of the panels.
(TIF)

S1 File Complete lists of NPM and NPM-MLF1 interacting proteins identified by LC-MS/MS analyses. (XLSX)

S2 File Complete lists of factors involved in transcriptional regulation identified by the LC-MS/MS analyses. (XLSX)

S3 File Complete lists of NPM and NPM-MLF1 interacting proteins separated by FPLC and regrouped in three peaks, identified by the LC-MS/MS analyses. (XLSX)

S4 File Complete list of genes with Log2 Fold change expression variations ≥ 1.5 in the NPM^KD^ (shNPM), identified by AmpliSeq analysis. (XLSX)
