# Supplementary material for: NPM and NPM-MLF1 interact with chromatin remodeling complexes and influence their recruitment to specific genes
Source: PLoS Genet. 2019 Nov 1;15(11):e1008463. doi: 10.1371/journal.pgen.1008463 (PMC6853375; doi:10.1371/journal.pgen.1008463)
Supplement: S1 Table — (DOCX) [file pgen.1008463.s001.docx]

| **Antibodies** | **References** | **Compagnies** | **Applications** |
| --- | --- | --- | --- |
| CHD4 | ab70469 | Abcam | Co-IP / ChIP |
| Mi2 (CHD3/CHD4) | sc-11378 | Santa-Cruz | Western Blot |
| MTA2 | sc-9447 | Santa-Cruz | Western Blot |
| MBD3 | ab157464 | Abcam | Western Blot |
| HDAC1 | sc-81598 | Santa-Cruz | Western Blot |
| BRG1 | sc-10768 | Santa-Cruz | Western Blot |
| BAZ1B/WSTF | sc-28426 | Santa-Cruz | Western Blot |
| SNF2H/SMARCA5 | ab3749 | Abcam | Co-IP /Western Blot |
| SNF2L/SMARCA1 | ab37003 | Abcam | Western Blot |
| BAF155 | sc-365505 | Santa-Cruz | Western Blot |
| HA | sc-805 | Santa-Cruz | Western Blot/IF |
| APE1 | ab92744 | Abcam | Western Blot |
| NPM | 32-5200 | Invitrogen | Western Blot / IF (Suppl Fig S1D) |
| CDK9 | sc-484 | Santa-Cruz | Co-IP/Western Blot |
| α-Tubulin | sc-23948 | Santa-Cruz | Western Blot |
| H3 | 06-755 | Millipore | Western Blot |
| H3K4me3 | ab8580 | Abcam | ChIP |
| H3K27me3 | 17-622 | Millipore | ChIP |
| H3K79me3 | ab2621 | Abcam | ChIP |
| H3K36me3 | 17-10032 | Millipore | ChIP |
| H3Ac | 06-599 | Millipore | ChIP |
| Pol II | sc-899 | Santa-Cruz | ChIP |
| TFIID | sc-273 | Santa-Cruz | ChIP |
| Ser2P-Pol II | 3E10 | Chromoteck | ChIP |
| HA | 3724 | Cell Signaling | ChIP |
| NPM | sc-271737 | Santa-Cruz | IF (Suppl Fig S1B) |
| Alexa fluor 594 anti rabbit | 111-585-144 | Jackson | IF |
| Alexa Fluor 488 anti mouse | 115-545-146 | Jackson | IF |
| Normal IgG |  | Millipore | Co-IP/Western Blot/ChIP |

**Supplementary S1 Table: List of antibodies**
