# Supplementary material for: NPM and NPM-MLF1 interact with chromatin remodeling complexes and influence their recruitment to specific genes
Source: PLoS Genet. 2019 Nov 1;15(11):e1008463. doi: 10.1371/journal.pgen.1008463 (PMC6853375; doi:10.1371/journal.pgen.1008463)
Supplement: S2 Table — (DOCX) [file pgen.1008463.s002.docx]

| **Gene/region** | **Sequence (Forward)** | **Sequence (Reverse)** | **Application** |
| --- | --- | --- | --- |
| CARD9 | TGCACCAGGAGCAGGTTTTG | TTTCTGCATCTTCCTGAGGGC | RT-qPCR |
| SPARC | ATTGACGGGTACCTCTCCCA | GAAAAAGCGGGTGGTGCAAT | RT-qPCR |
| MS4A3 | TACTTCTGTCTACCAGCCCAT | GTTCCTGAACTACAGAACCCA | RT-qPCR |
| NPM | GTACAGCCAACGGTTTCCCTT | CTCTGCATCTTCCTCCACAGC | RT-qPCR |
| GAPDH | GTCGGAGTCAACGGATT | AAGCTTCCCGTTCTCAG | RT-qPCR |
| CARD9 TSS | CAGCGGACTTCCACTTCCGT | CCTGGTGACCTGCTTTCTGATT | ChIP-qPCR |
| CARD9 ORF | CGACCCAACCCTTCTCTGTGA | CCTGAACTGCTCGTGTGCC | ChIP-qPCR |
| SPARC TSS | GTGAGTCGGTTTAGGCAGCA | TCTCCCATAGCCAAAGCTGTC | ChIP-qPCR |
| SPARC ORF | AACTTTTGGGAGCACGGACT | GTCCCTAGAGCCCCTGAGAA | ChIP-qPCR |
| MS4A3 TSS | GTCTGTGGAGGCTTCCGTTA | AGTCCACCTTGTCGCAAGTA | ChIP-qPCR |
| Pax6 | AAGCAATGATGTTCTGCCAGC | CTGCCATCCTGCTGACTTTCC | ChIP-qPCR |

**Supplementary S2 Table: Primer sequence table**
