# Supplementary figures and images for: NPM and NPM-MLF1 interact with chromatin remodeling complexes and influence their recruitment to specific genes
Source: PLoS Genet. 2019 Nov 1;15(11):e1008463. doi: 10.1371/journal.pgen.1008463 (PMC6853375; doi:10.1371/journal.pgen.1008463)

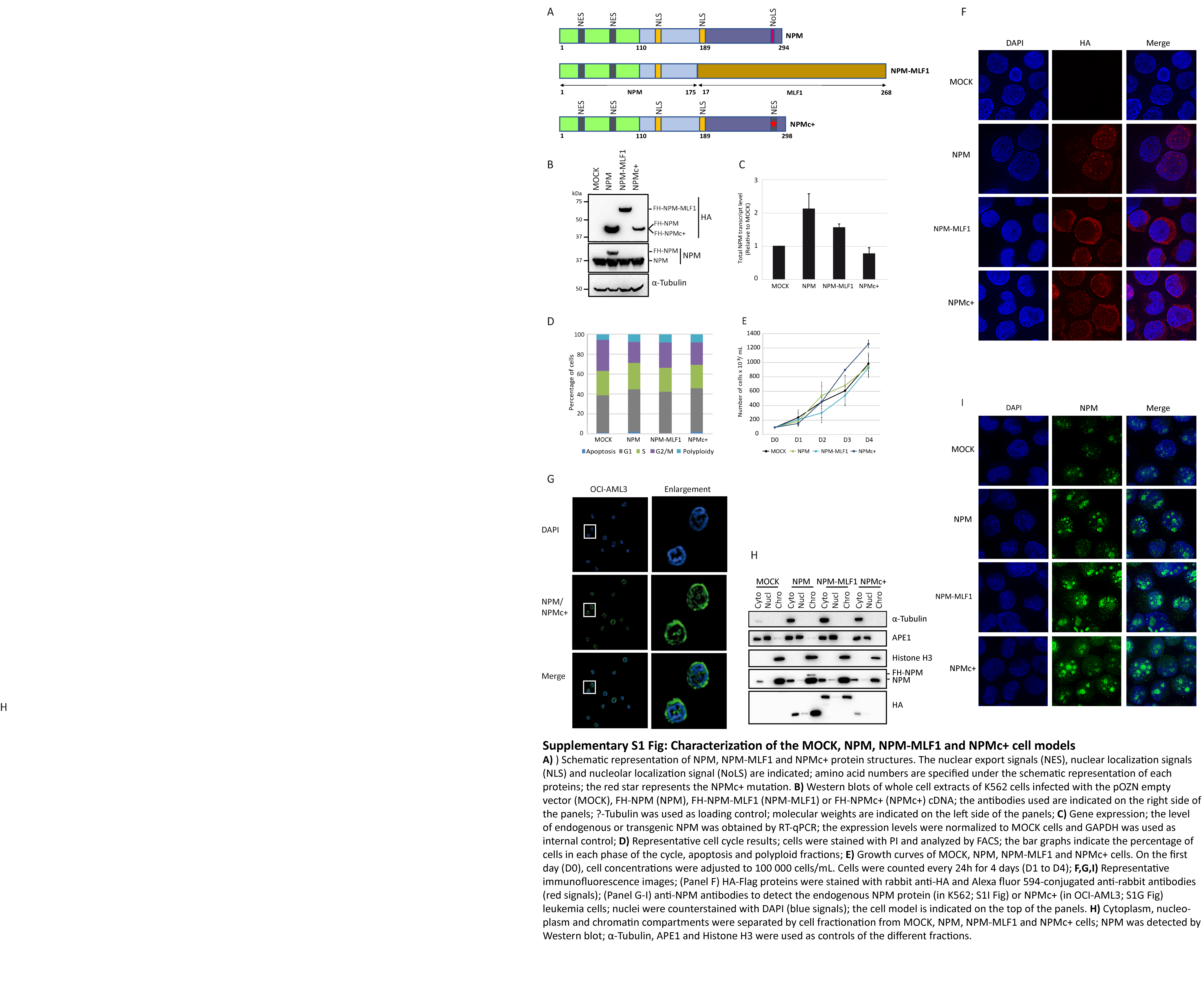

Supplement: S1 Fig — A) Schematic representation of NPM, NPM-MLF1 and NPMc+ protein structures. The nuclear export signals (NES), nuclear localization signals (NLS) and nucleolar localization signal (NoLS) are indicated; amino acid numbers are specified under the schematic representation of each proteins; the red star represents the NPMc+ mutation. B) Western blots of whole cell extracts of K562 cells infected with the pOZN empty vector (MOCK), FH-NPM (NPM), FH-NPM-MLF1 (NPM-MLF1) or FH-NPMc+ (NPMc+) cDNA; the antibodies used are indicated on the right side of the panels; α-Tubulin was used as loading control; molecular weights are indicated on the left side of the panels; C) Gene expression; the level of endogenous or transgenic NPM was obtained by RT-qPCR; the expression levels were normalized to MOCK cells and GAPDH was used as internal control; D) Representative cell cycle results; cells were stained with PI and analyzed by FACS; the bar graphs indicate the percentage of cells in each phase of the cycle, apoptosis and polyploid fractions; E) Growth curves of MOCK, NPM, NPM-MLF1 and NPMc+ cells. On the first day (D0), cell concentrations were adjusted to 100 000 cells/mL. Cells were counted every 24h for 4 days (D1 to D4); F,G,I) Representative immunofluorescence images; (Panel F) HA-Flag proteins were stained with rabbit anti-HA and Alexa fluor 594-conjugated anti-rabbit antibodies (red signals); (Panel G-I) anti-NPM antibodies to detect the endogenous NPM protein (in K562; S1I Fig) or NPMc+ (in OCI-AML3; S1G Fig) leukemia cells; nuclei were counterstained with DAPI (blue signals); the cell model is indicated on the top of the panels. H) Cytoplasm, nucleoplasm and chromatin compartments were separated by cell fractionation from MOCK, NPM, NPM-MLF1 and NPMc+ cells; NPM was detected by Western blot; α-Tubulin, APE1 and Histone H3 were used as controls of the different fractions. (TIF) [file pgen.1008463.s003.tif]

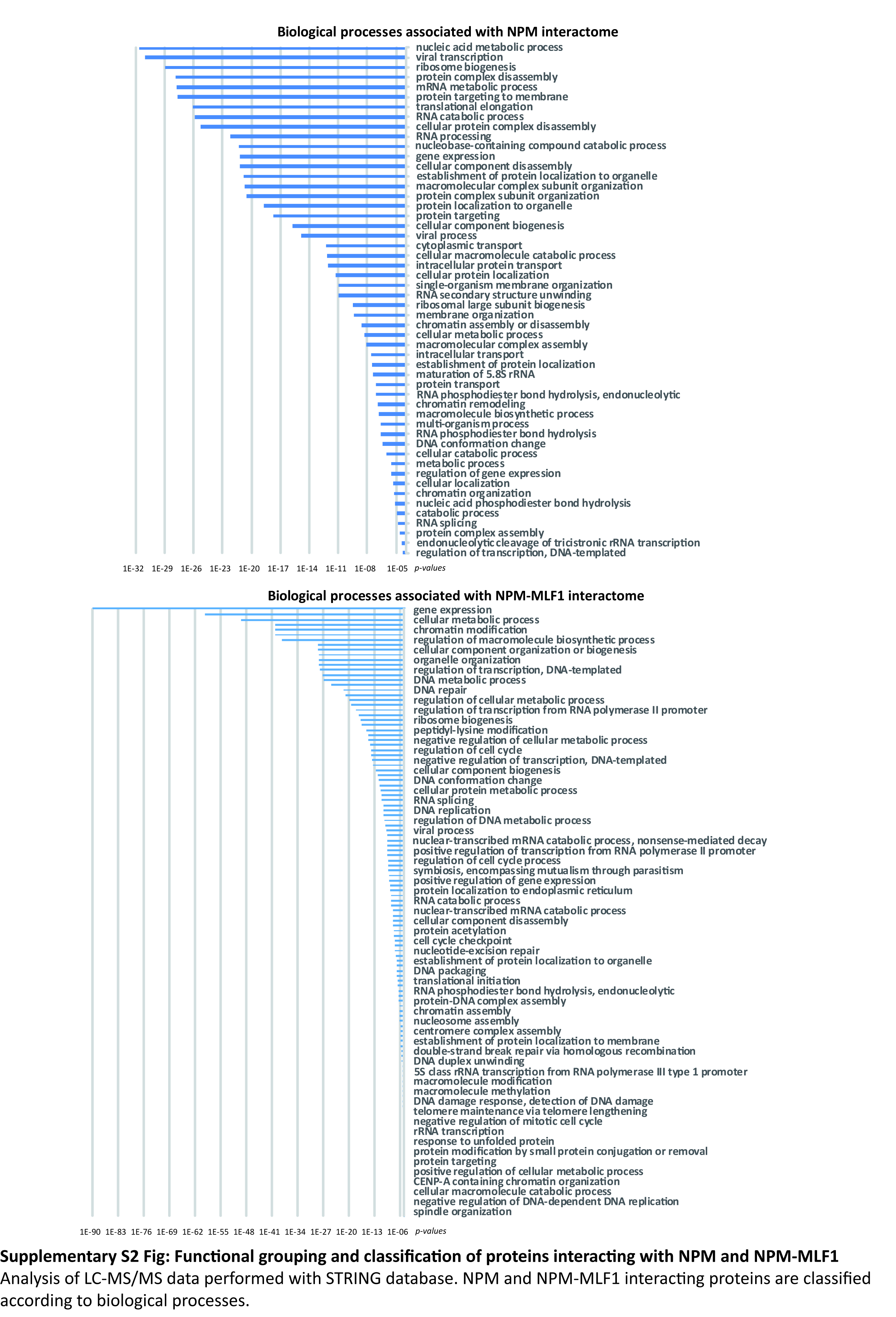

Supplement: S2 Fig — Analysis of LC-MS/MS data performed with STRING database. NPM and NPM-MLF1 interacting proteins are classified according to biological processes. (TIF) [file pgen.1008463.s004.tif]

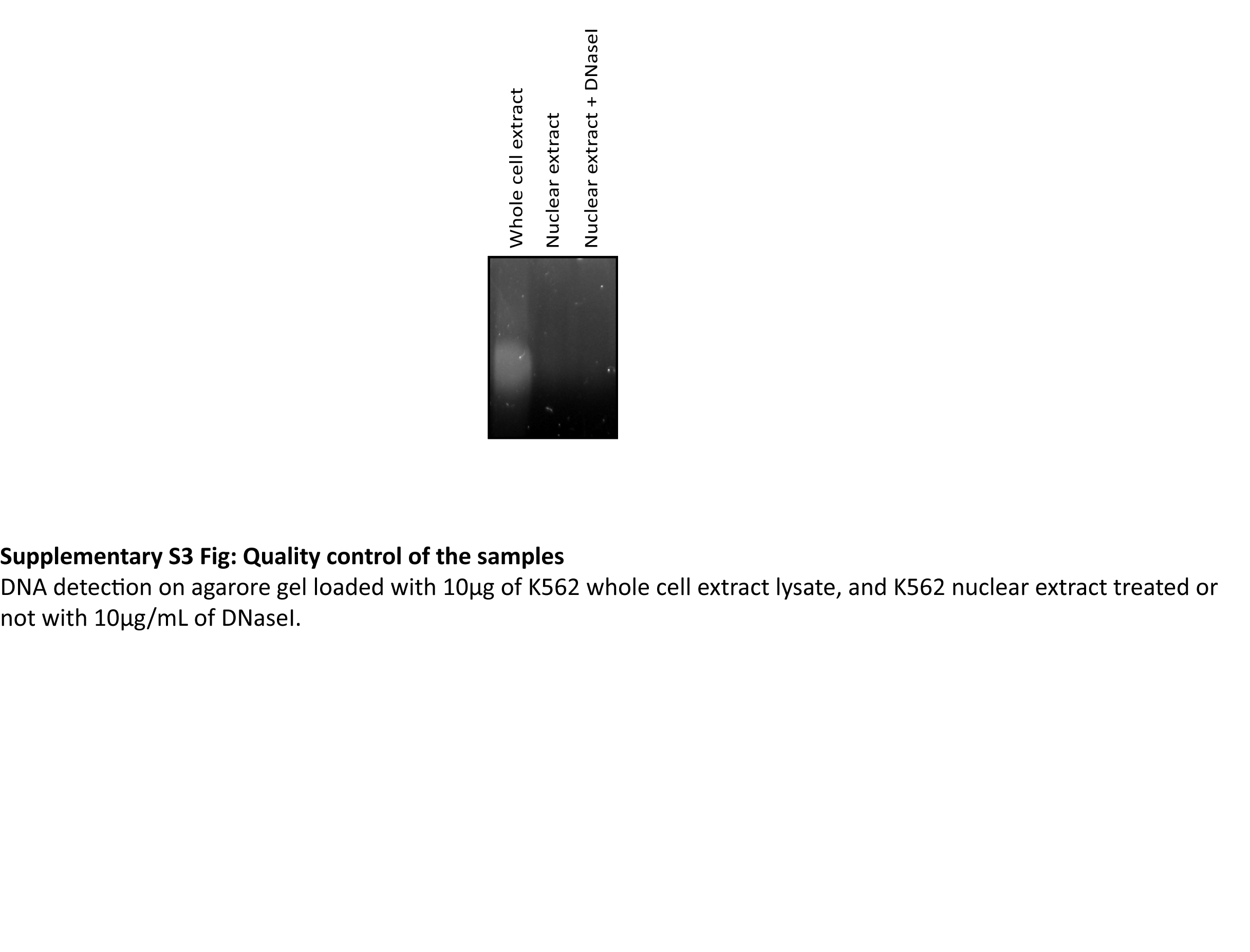

Supplement: S3 Fig — DNA detection on agarore gel loaded with 10μg of K562 whole cell extract lysate, and K562 nuclear extract treated or not with 10μg/mL of DNaseI. (TIF) [file pgen.1008463.s005.tif]

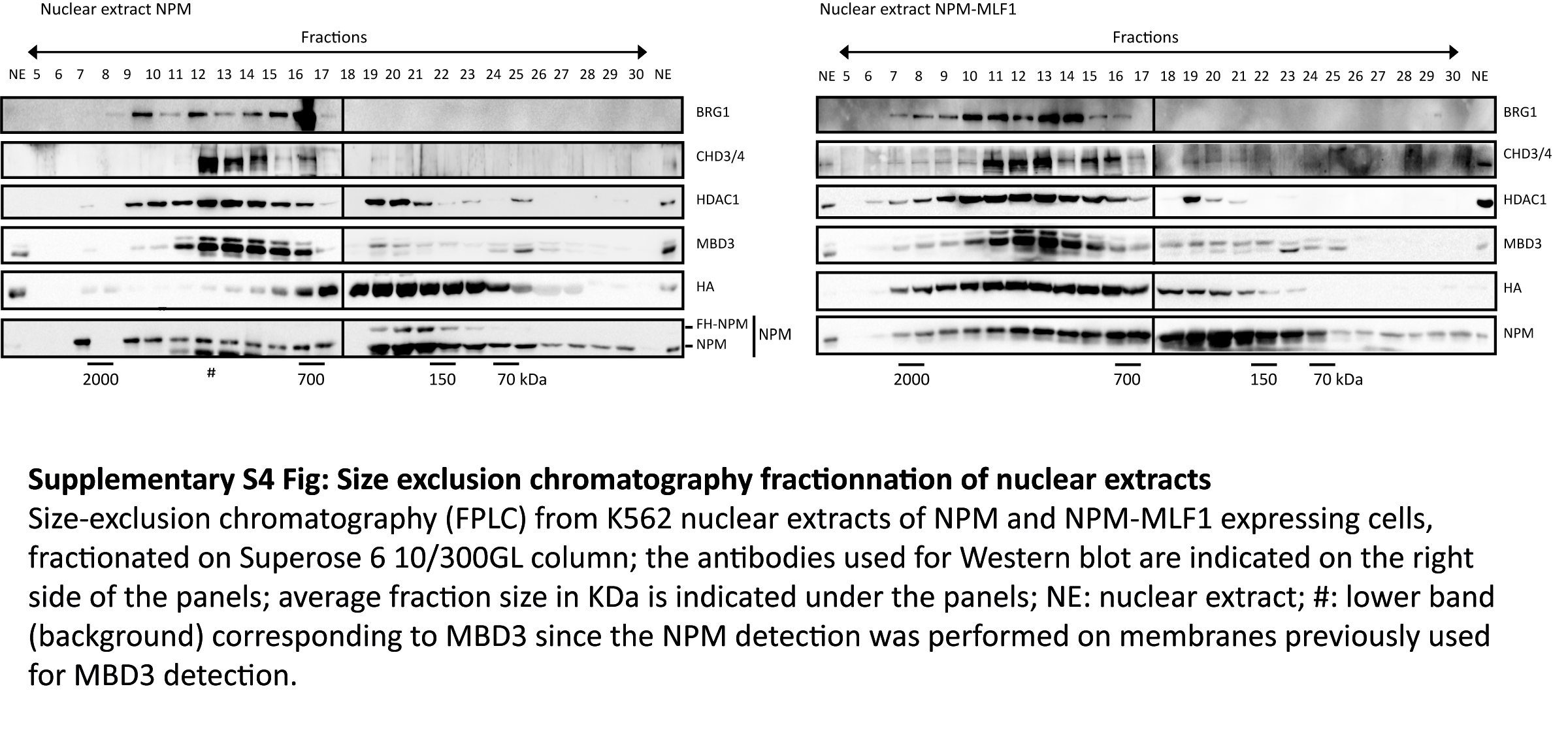

Supplement: S4 Fig — Size-exclusion chromatography (FPLC) from K562 nuclear extracts of NPM and NPM-MLF1 expressing cells, fractionated on Superose 6 10/300GL column; the antibodies used for Western blot are indicated on the right side of the panels; average fraction size in KDa is indicated under the panels; NE: nuclear extract; #: lower band (background) corresponding to MBD3 since the NPM detection was performed on membranes previously used for MBD3 detection. (TIF) [file pgen.1008463.s006.tif]

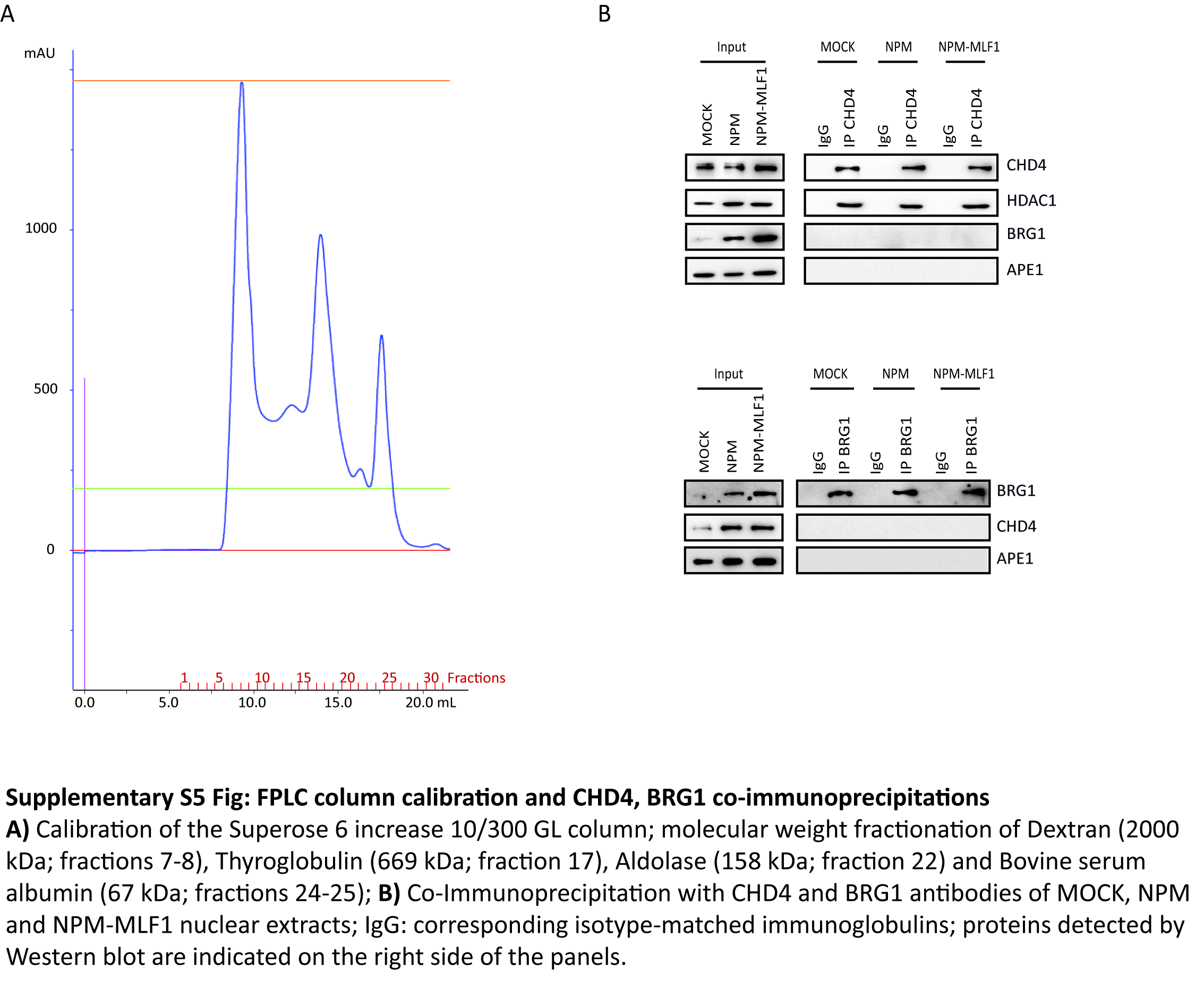

Supplement: S5 Fig — A) Calibration of the Superose 6 increase 10/300 GL column; molecular weight fractionation of Dextran (2000 kDa; fractions 7–8), Thyroglobulin (669 kDa; fraction 17), Aldolase (158 kDa; fraction 22) and Bovine serum albumin (67 kDa; fractions 24–25); B) Co-Immunoprecipitation with CHD4 and BRG1 antibodies of MOCK, NPM and NPM-MLF1 nuclear extracts; IgG: corresponding isotype-matched immunoglobulins; proteins detected by Western blot are indicated on the right side of the panels. (TIF) [file pgen.1008463.s007.tif]

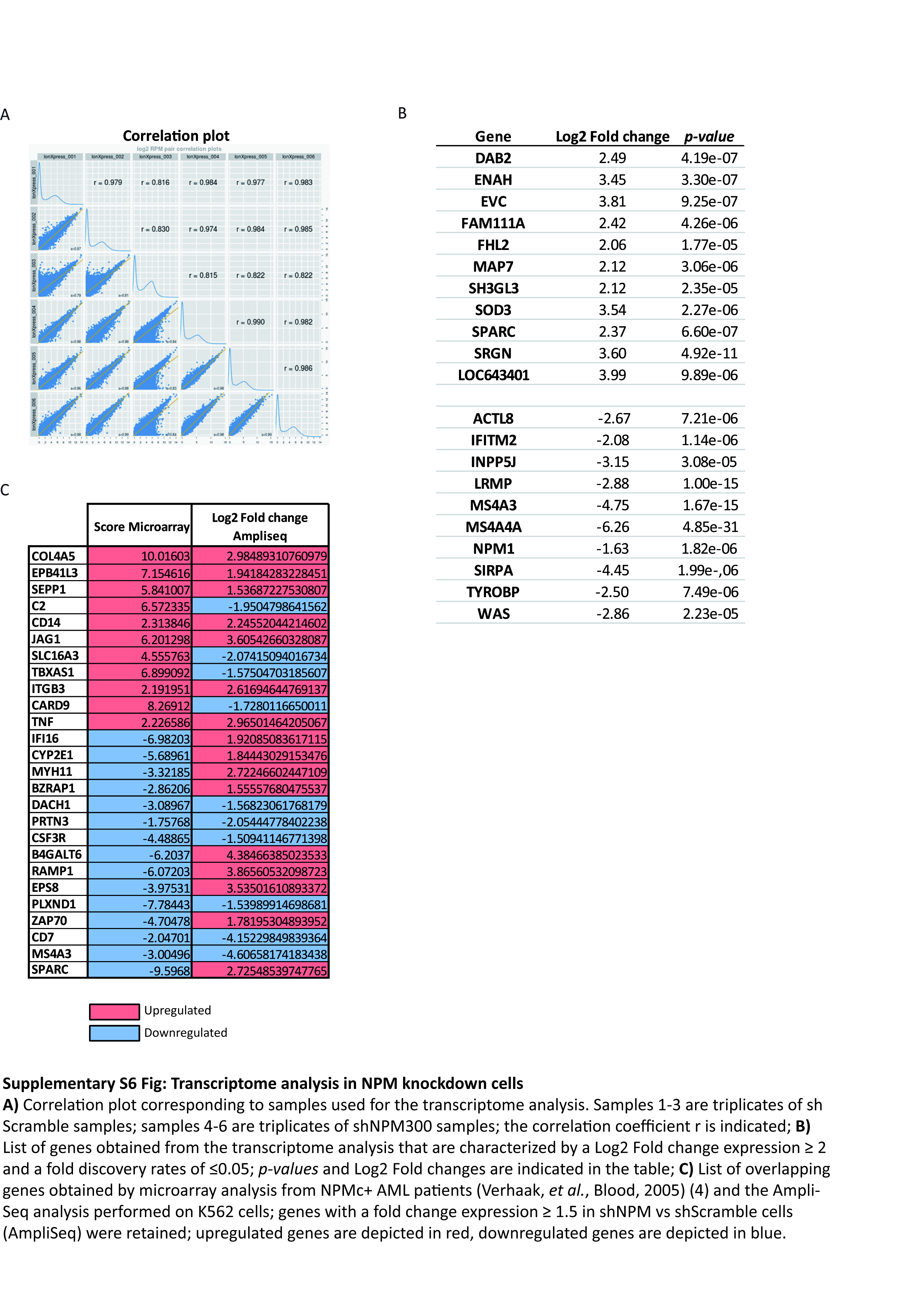

Supplement: S6 Fig — A) Correlation plot corresponding to samples used for the transcriptome analysis. Samples 1–3 are triplicates of shScramble samples; samples 4–6 are triplicates of shNPM300 samples; the correlation coefficient r is indicated; B) List of genes obtained from the transcriptome analysis that are characterized by a Log2 Fold change expression ≥2 and a fold discovery rates of ≤0.05; p-values and Log2 Fold changes are indicated in the table; C) List of overlapping genes obtained by microarray analysis from NPMc+ AML patients (Verhaak, et al., Blood, 2005) (4) and the AmpliSeq analysis performed on K562 cells; genes with a fold change expression ≥ 1.5 in shNPM vs shScramble cells (AmpliSeq) were retained; upregulated genes are depicted in red, downregulated genes are depicted in blue. (TIF) [file pgen.1008463.s008.tif]

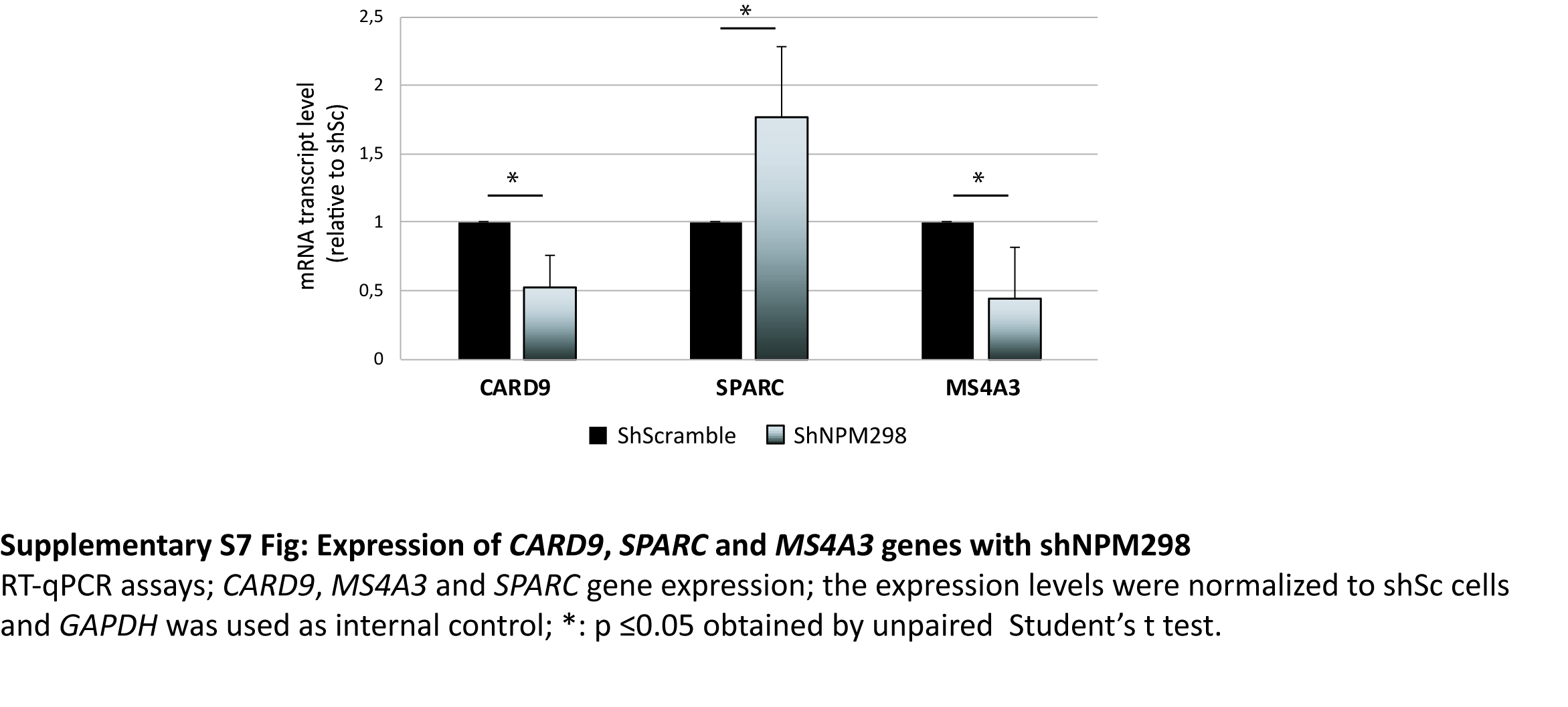

Supplement: S7 Fig — RT-qPCR assays; CARD9, MS4A3 and SPARC gene expression; the expression levels were normalized to shSc cells and GAPDH was used as internal control; *: p ≤0.05 obtained by unpaired Student’s t test. (TIF) [file pgen.1008463.s009.tif]

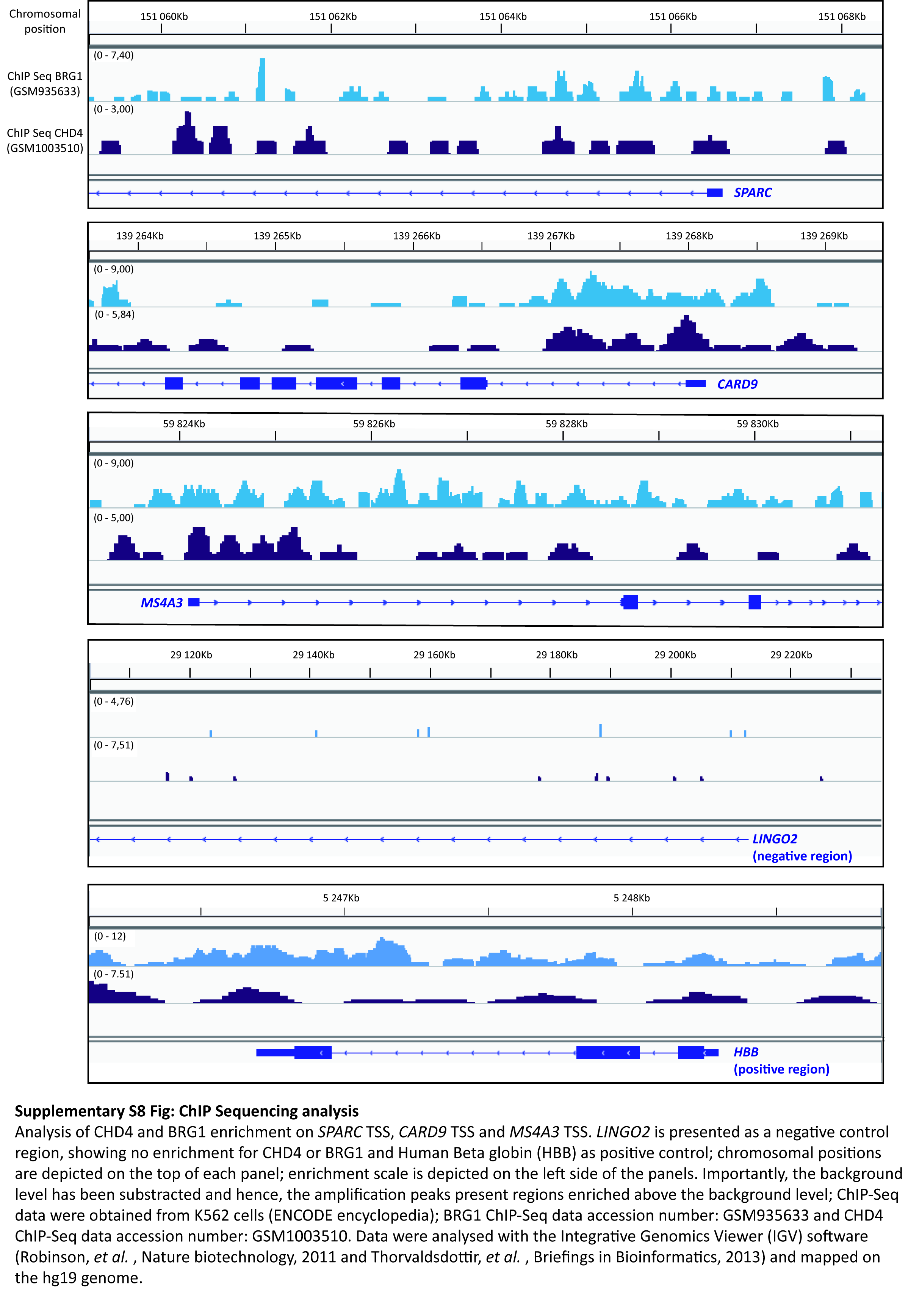

Supplement: S8 Fig — Analysis of CHD4 and BRG1 enrichment on SPARC TSS, CARD9 TSS and MS4A3 TSS. LINGO2 is presented as a negative control region, showing no enrichment for CHD4 or BRG1 and Human Beta globin (HBB) as positive control; chromosomal positions are depicted on the top of each panel; enrichment scale is depicted on the left side of the panels. Importantly, the background level has been substracted and hence, the amplification peaks present regions enriched above the background level; ChIP-Seq data were obtained from K562 cells (ENCODE encyclopedia); BRG1 ChIP-Seq data accession number: GSM935633 and CHD4 ChIP-Seq data accession number: GSM1003510. Data were analysed with the Integrative Genomics Viewer (IGV) software (Robinson, et al., Nature biotechnology, 2011 and Thorvaldsdottir, et al., Briefings in Bioinformatics, 2013) and mapped on the hg19 genome. (TIF) [file pgen.1008463.s010.tif]

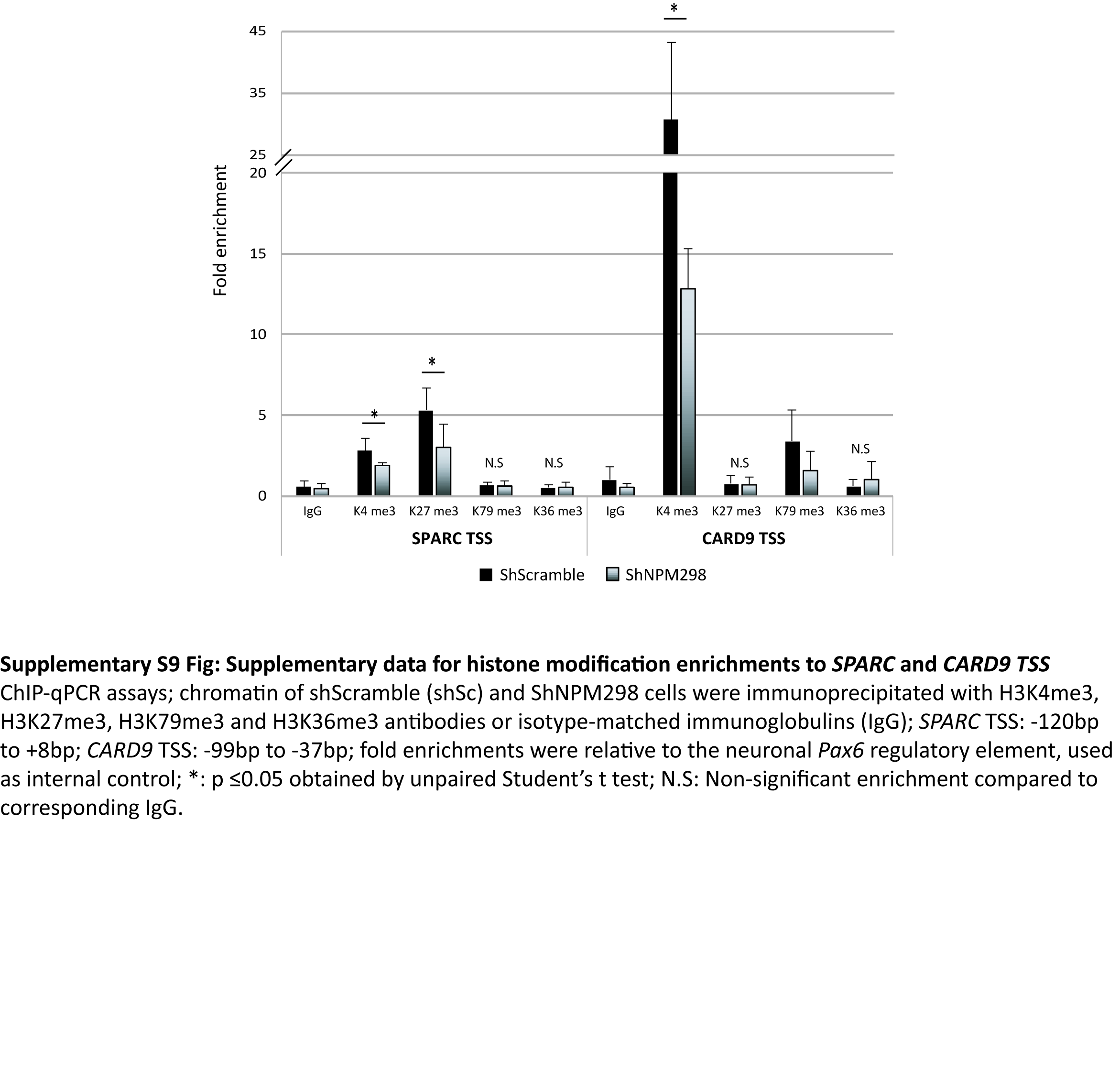

Supplement: S9 Fig — ChIP-qPCR assays; chromatin of shScramble (shSc) and ShNPM298 cells were immunoprecipitated with H3K4me3, H3K27me3, H3K79me3 and H3K36me3 antibodies or isotype-matched immunoglobulins (IgG); SPARC TSS: -120bp to +8bp; CARD9 TSS: -99bp to -37bp; fold enrichments were relative to the neuronal Pax6 regulatory element, used as internal control; *: p ≤0.05 obtained by unpaired Student’s t test; N.S: Non-significant enrichment compared to corresponding IgG. (TIF) [file pgen.1008463.s011.tif]

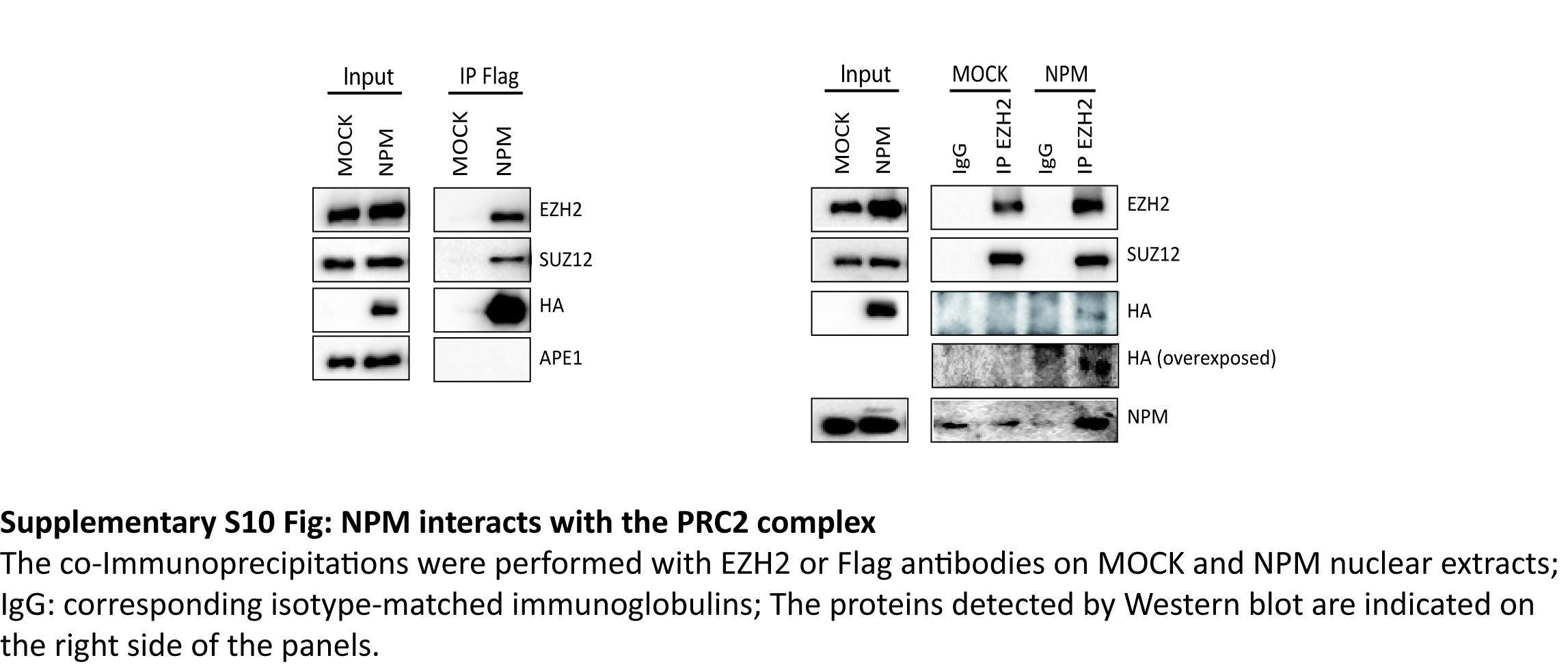

Supplement: S10 Fig — The co-immunoprecipitations were performed with EZH2 or Flag antibodies on MOCK and NPM nuclear extracts; IgG: corresponding isotype-matched immunoglobulins; The proteins detected by Western blot are indicated on the right side of the panels. (TIF) [file pgen.1008463.s012.tif]

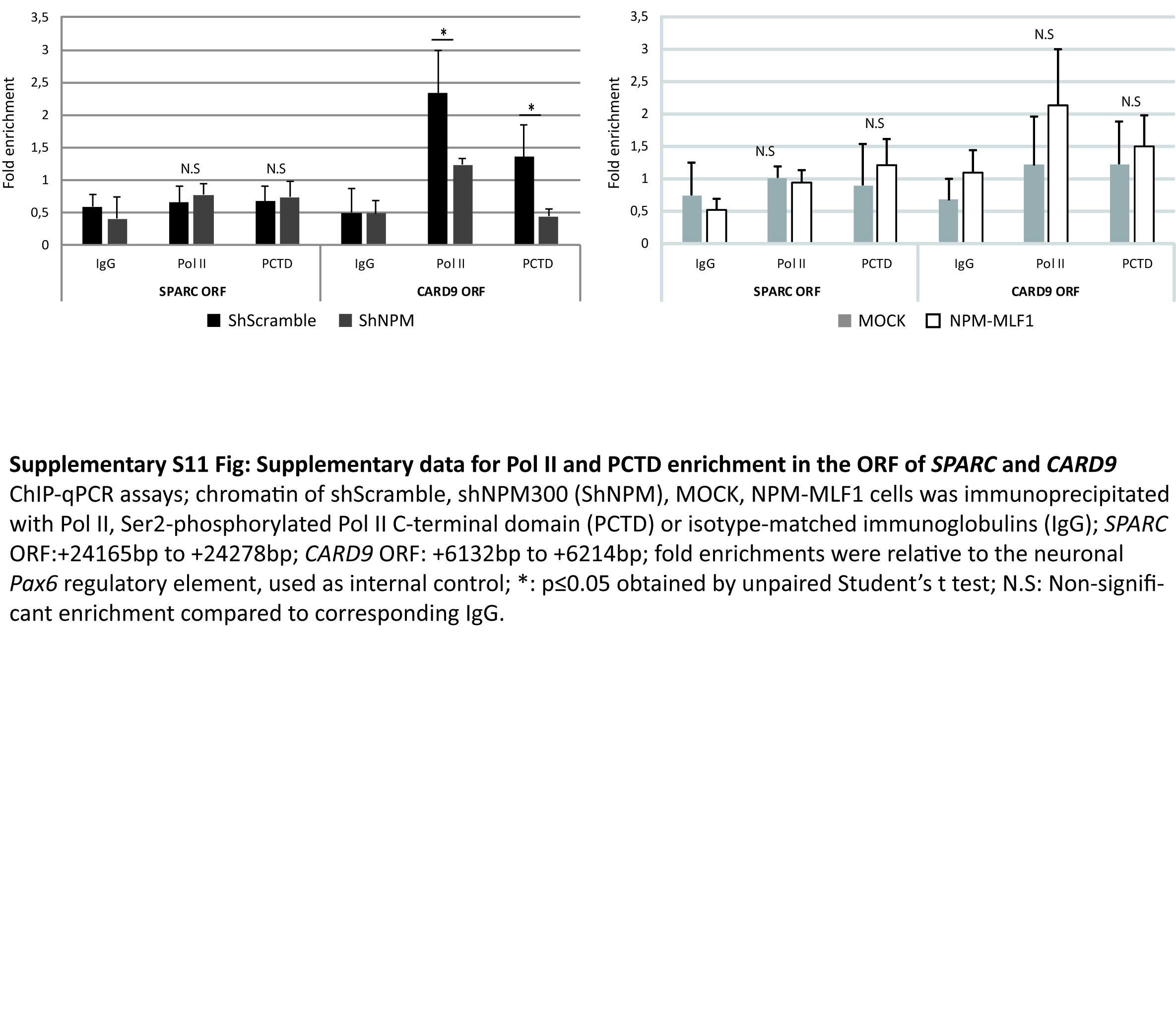

Supplement: S11 Fig — ChIP-qPCR assays; chromatin of shScramble, shNPM300 (ShNPM), MOCK, NPM-MLF1 cells was immunoprecipitated with Pol II, Ser2-phosphorylated Pol II C-terminal domain (PCTD) or isotype-matched immunoglobulins (IgG); SPARC ORF: +24165bp to +24278bp; CARD9 ORF: +6132bp to +6214bp; fold enrichments are relative to the neuronal Pax6 regulatory element, used as internal control; *: p ≤0.05 obtained by unpaired Student’s t test; N.S: Non-significant enrichment compared to corresponding IgG. (TIF) [file pgen.1008463.s013.tif]

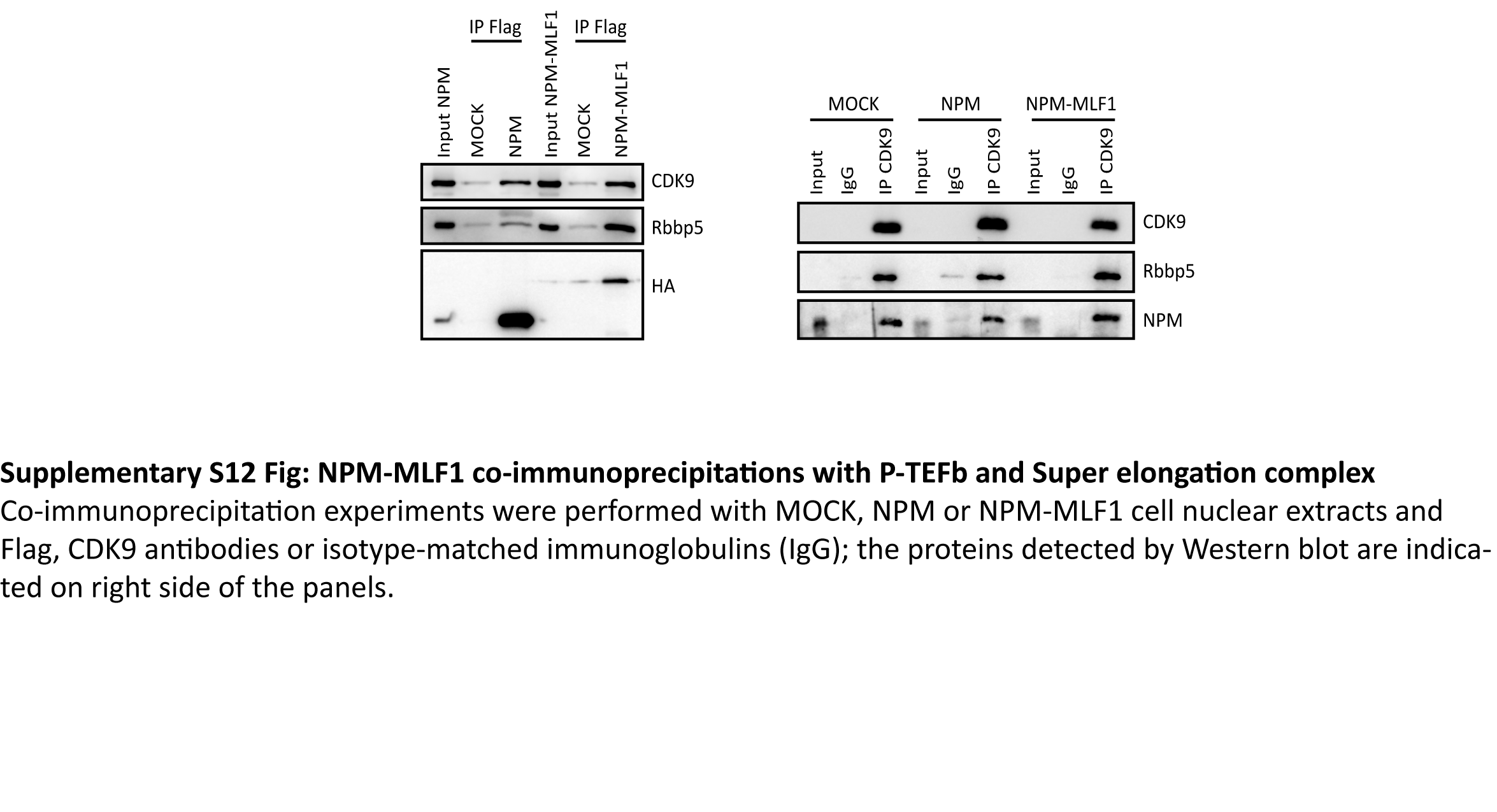

Supplement: S12 Fig — Co-immunoprecipitation experiments were performed with MOCK, NPM or NPM-MLF1 cell nuclear extracts, and with Flag, CDK9 antibodies or isotype-matched immunoglobulins (IgG); the proteins detected by Western blot are indicated on right side of the panels. (TIF) [file pgen.1008463.s014.tif]

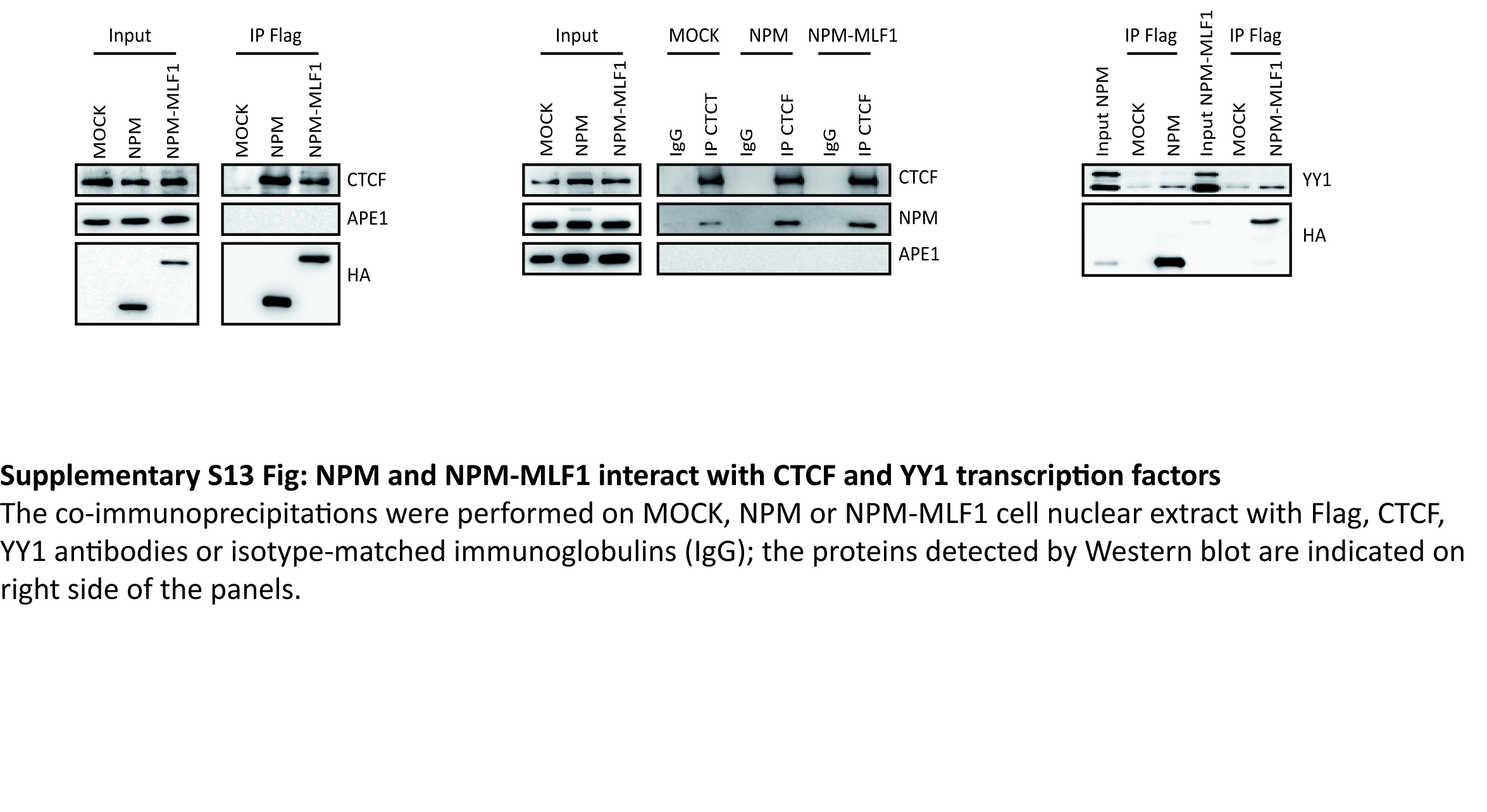

Supplement: S13 Fig — The co-immunoprecipitations were performed on MOCK, NPM or NPM-MLF1 cell nuclear extracts with Flag, CTCF, YY1 antibodies or isotype-matched immunoglobulins (IgG); the proteins detected by Western blot are indicated on right side of the panels. (TIF) [file pgen.1008463.s015.tif]
